# The co-occurrence of multimorbidity and polypharmacy among middle-aged and older adults in Canada: A cross-sectional study using the Canadian Longitudinal Study on Aging (CLSA) and the Canadian Primary Care Sentinel Surveillance Network (CPCSSN)

**DOI:** 10.1371/journal.pone.0312873

**Published:** 2025-01-15

**Authors:** Kathryn Nicholson, Jennifer Salerno, Sayem Borhan, Benoit Cossette, Dale Guenter, Meredith Vanstone, John Queenan, Michelle Greiver, Michelle Howard, Amanda L. Terry, Tyler Williamson, Lauren E. Griffith, Martin Fortin, Saverio Stranges, Dee Mangin

**Affiliations:** 1 Department of Epidemiology & Biostatistics, Western University, London, Ontario, Canada; 2 Department of Family Medicine, McMaster University, Hamilton, Ontario, Canada; 3 Department of Health Research Methods, Evidence and Impact, McMaster University, Hamilton, Ontario, Canada; 4 Faculté de médecine et des sciences de la santé, Université de Sherbrooke, Sherbrooke, Québec, Canada; 5 Department of Family Medicine, Queen’s University, Kingston, Ontario, Canada; 6 Department of Family and Community Medicine, University of Toronto, Toronto, Ontario, Canada; 7 Department of Family Medicine, Western University, London, Ontario, Canada; 8 Interfaculty Program in Public Health, Western University, London, Ontario, Canada; 9 Department of Community Health Sciences, University of Calgary, Calgary, Alberta, Canada; 10 Department of Family Medicine and Emergency Medicine, Université de Sherbrooke, Sherbrooke, Québec, Canada; 11 Department of Medicine, Western University, London, Ontario, Canada; 12 Department of Clinical Medicine and Surgery, University of Naples Federico II University, Naples, Italy; 13 Department of Primary Care and Clinical Simulation, University of Otago, Christchurch, New Zealand; Bushehr University of Medical Sciences, ISLAMIC REPUBLIC OF IRAN

## Abstract

**Background:**

There is an increasing prevalence of multiple conditions (multimorbidity) and multiple medications (polypharmacy) across many populations. Previous literature has focused on the prevalence and impact of these health states separately, but there is a need to better understand their co-occurrence.

**Methods and findings:**

This study reported on multimorbidity and polypharmacy among middle-aged and older adults in two national datasets: the Canadian Longitudinal Study on Aging (CLSA) and the Canadian Primary Care Sentinel Surveillance Network (CPCSSN). Using consistent methodology, we conducted a cross-sectional analysis of CLSA participants and CPCSSN patients aged 45 to 85 years as of 2015. When multimorbidity was defined as two or more conditions, the prevalence was 66.7% and 52.0% in the CLSA and CPCSSN cohorts, respectively. The prevalence of polypharmacy was 14.9% in the CLSA cohort and 22.6% in the CPCSSN cohort when defined as five or more medications. Using the same cut-points, the co-occurrence of multimorbidity and polypharmacy was similar between the two cohorts (CLSA: 14.3%; CPCSSN: 13.5%). Approximately 20% of older adults (65 to 85 years) were living with both multimorbidity and polypharmacy (CLSA: 21.4%; CPCSSN: 18.3%), as compared to almost 10% of middle-aged adults (45 to 64 years) living with this co-occurrence (CLSA: 9.2%; CPCSSN: 9.9%). Across both cohorts and age groups, females had consistently higher estimates of multimorbidity, polypharmacy and the co-occurrence of multimorbidity and polypharmacy.

**Conclusions:**

This study found that multimorbidity and polypharmacy are not interchangeable in understanding population health needs. Approximately one in five older adults in the CLSA and CPCSSN cohorts were living with both multimorbidity and polypharmacy, double the proportion in the younger cohorts. This has implications for future research, as well as health policy and clinical practice, that aim to reduce the occurrence and impact of multimorbidity and unnecessary polypharmacy to enhance the well-being of aging populations.

## Introduction

The advances of public health and clinical medicine have allowed individuals to live longer, but similar increases in quality of life have not followed [1]. While an increased lifespan represents an important achievement, there is now a need to ensure that health span (quality of life) extends along with this lifespan (quantity of life). The accumulation of multiple conditions (multimorbidity) within an individual, as well as the multiple medications (polypharmacy) used to manage or treat these conditions, have become significant challenges for public health, health care systems, health care providers and individuals living with these health states [2–7]. Multimorbidity and polypharmacy are often strongly associated [8–10]. A powerful driver of this is the current clinical guidelines framework that is typically used in providing care [2, 4, 7]. These have been largely developed for single conditions in isolation, yet do not sufficiently account for common scenarios in which management of multiple conditions may be required at one time [6, 11–16]. Indeed, addressing the use of multiple prescribed medications and minimizing the potential occurrence of harm and treatment burden have been acknowledged as key tasks for improvement in the clinical management of multimorbidity [3, 4, 6, 17–21].

To date, there have been a number of reviews conducted to describe the measurement, prevalence and factors associated with the occurrence of multimorbidity and polypharmacy separately in various settings [4, 22–38]. Living with multimorbidity and polypharmacy can create substantial challenges that extend beyond the conditions and symptoms themselves [6, 34, 39] including increased risk of medication interactions [3, 4, 15, 34, 40, 41], barriers to self-care and self-management [42–45], reduced quality of life [32, 35, 43, 46, 47], higher healthcare costs [48–52] and difficulty coordinating health care services [7, 43, 53–55]. Polypharmacy also carries the potential for inappropriate or problematic prescribing, particularly as the number of concomitant medications increases, if these are not managed and reviewed regularly by health care providers, patients and caregivers [31, 56].

A recent systematic review was conducted to identify original research that reported on the prevalence of both multimorbidity and polypharmacy among adults or older adults in community or primary care settings [57]. The 87 included studies reported a prevalence of multimorbidity (defined as two or more conditions) that ranged from 4.8% to 93.1%, while the reported prevalence of polypharmacy (defined as five or more medications) ranged from 2.6% to 86.6% [57]. Of course, these substantial ranges in prevalence are partly due to the methodological heterogeneity and the lack of a consistent operational definition for these two concepts [4, 5, 30, 36–38], but this review also identified the lack of studies that reported on the prevalence of the co-occurrence of multimorbidity and polypharmacy within the same individuals (that is, instead of the calculation and reporting on multimorbidity and polypharmacy separately). Based on previous literature that has highlighted the higher risk of negative health outcomes (such as drug-drug interactions, drug-disease interactions and hospital admissions) for individuals living with both multimorbidity and polypharmacy, there is a need to identify and describe the prevalence of these concurrent health states in various populations.

However, there is no single set of data that represents the “gold standard” approach to reporting on multimorbidity and polypharmacy. For example, clinical and administrative data may capture medications that have been prescribed or dispensed, but health survey data may capture medications that are actually being taken regularly by individuals. Previous research has shown that self-reported data has reasonable levels of validity and accuracy when compared with clinical or administrative datasets, but this can vary by specific conditions [58–60]. Self-reported medication data show reasonable accuracy when compared to prescribing records, particularly for medications that are being used to treat chronic conditions [61–63]. For both conditions and medications, self-reported data can be influenced by factors such as age, gender, health literacy, recall bias and social desirability bias. The accuracy and validity of clinical data for conditions and medications requires consistent and complete documentation by primary care providers, as well as data processing and standardization, particularly because these data are documented for clinical and not research purposes [64–67]. Linked datasets that combine multiple sources of information can address the limitations of individual sources, but when this is not available, complementary data sources can be used to provide insight into this complex issue.

This study aimed to report on the co-occurrence of multimorbidity and polypharmacy among middle-aged and older adults using consistent methodology in two national datasets in Canada. Our objectives were to: 1) determine the prevalence of multimorbidity and polypharmacy (individually and concurrently) in a national community-based cohort study composed of self-reported data; and 2) determine the prevalence of multimorbidity and polypharmacy (individually and concurrently) in a national electronic medical record dataset composed of primary care data.

## Methods

### Data sources

The Canadian Longitudinal Study on Aging (CLSA) is a national prospective longitudinal cohort study collecting data from more than 50,000 community-dwelling participants who were aged 45 to 85 years at recruitment [68–70]. These data are collected either through telephone interviews (Tracking cohort) or directly in-person at the participants’ home or at data collection sites (Comprehensive cohort) that are located across the country with follow-up data collection occurring every three years [68–70]. The Comprehensive cohort was composed of participants who lived within 25 to 50 km of one of the eleven data collection sites located in seven provinces and baseline data collection was completed as of 2015 [68–70]. Due to the in-person and more in-depth data collection, the Comprehensive cohort may have an underrepresentation of individuals with lowers levels of literacy in French or English, memory impairment or mobility issues [69, 70]. However, this cohort had information recorded for both conditions and medications. To capture multimorbidity, the participants were asked “Has a doctor ever told you that you have…” for each of the conditions in the multimorbidity operational definition. To capture polypharmacy, the participants were asked to present all regularly scheduled or taken medications, which were then mapped to the World Health Organization (WHO) Anatomical Therapeutic Chemical (ATC) classification [68, 71]. The CLSA data were initially accessed on 02/02/2022 and no individual participants could be identified within the dataset used for research purposes.

The Canadian Primary Care Sentinel Surveillance Network (CPCSSN) is a national electronic medical record (EMR) database collecting longitudinal data from more than 1.5 million community-dwelling patients who are receiving care from a participating primary care site [72–75]. These data are collected from all patient encounters with a primary care provider who has consented to contributing de-identified data to the centralized dataset, which are then cleaned and coded by algorithms to facilitate use [73, 76]. These data are compiled into this centralized dataset from ten regional networks with primary care sites from inner-city, urban, suburban, small town and rural settings [73]. This database has been shown to have a reasonable level of representativeness and is continuously working to expand its coverage [73, 77]. A two-year contact group was used to identify the sample of patients who had at least one primary care encounter over a two-year timeframe (January 1, 2014 to December 31, 2015) and who were more likely to have up-to-date documentation [73]. The conditions and medications that were documented between 2010 and 2015 were used to identify multimorbidity and polypharmacy. More specifically, the date on which a condition was diagnosed or a medication was prescribed was between 2010 and 2015 for reasonable levels of data quality and completeness [73]. The CPCSSN data were initially accessed on 18/07/2022 and no individual patients could be identified within the dataset used for research purposes.

### Study samples

For the purpose of comparison between the two datasets, the sample for this study focused on CLSA participants and CPCSSN patients aged 45 to 85 years as of 2015. In the CLSA cohort, this included individuals who were recruited at baseline and had complete data on birth year and sex. In the CPCSSN cohort, this included individuals who had at least one primary care encounter over a two-year period (2014 to 2015) who had complete data on birth year and sex. The denominator for the CLSA dataset was all participants aged 45 to 85 years as of 2015 in the Comprehensive baseline cohort (N = 30097) and the denominator for the CPCSSN dataset was all patients aged 45 to 85 years as of 2015 who had an encounter with their primary care provider between 2014 and 2015 (N = 597631). Each of these are unique participants and patients, and there is a possibility that the same individuals are included in the CLSA and CPCSSN datasets, but no current linkage can confirm this.

### Definition of multimorbidity

Multimorbidity was identified using a list of 18 conditions and two cut-points (two or more and three or more conditions). Although the original list consists of 20 conditions, 18 of the 20 conditions were used in the current study to align with the conditions captured in the CLSA dataset and the corresponding International Classification of Disease (ICD-9) codes that were applied in the CPCSSN dataset [78]. The list of conditions and the corresponding ICD-9 codes is presented in S1 Table.

### Definition of polypharmacy

Polypharmacy was identified using ATC Level 4 codes and two cut-points (five or more and ten or more medications). There was a total of 909 codes within Level 4 and if multiple medications were being taken within the same ATC Level 4, this was only counted once for a participant or patient (for example: multiple corticosteroids were only counted once as C05AA). The list of included ATC codes is presented in S2 Table.

### Statistical analysis

The mean number of conditions, mean number of medications and the distribution of participants or patients using the two cut-points of multimorbidity (two or more and three or more conditions) and polypharmacy (five or more and ten or more medications) were reported for the overall CLSA and CPCSSN cohorts, as well as stratified by age group (45 to 64 years or 65 to 85 years) and sex (female or male). More specifically, participants and patients were categorized with neither multimorbidity nor polypharmacy, multimorbidity only, polypharmacy only or both multimorbidity and polypharmacy. The prevalence of the individual conditions and medications that were included in the operational definitions of multimorbidity and polypharmacy were also reported for the overall CLSA and CPCSSN cohorts. As this study was focused on the comparison between two national cohorts using descriptive analyses, no weighting or multivariable analyses were conducted. Only participants and patients with a documented birth year and sex were included in the final samples for each cohort. Data management and data analyses were conducted using Stata SE 17.0 [79].

### Ethics approval

The research ethics approval was obtained from the Hamilton Integrated Research Ethics Board (HiREB) Project Number 14045.

## Results

### Overall samples

The characteristics and prevalence estimates for the overall CLSA and CPCSSN cohorts are presented in Table 1. In both cohorts, the majority of the sample was between 45 and 64 years of age (CLSA: 58.0%; CPCSSN: 57.2%) and female (CLSA: 50.9%; CPCSSN: 55.0%). The prevalence of MM2+ was 66.7% among CLSA participants and 52.0% among CPCSSN patients, while the prevalence of MM3+ was 46.1% in the CLSA cohort and 30.2% in the CPCSSN cohort. Among CLSA participants, the prevalence of PP5+ and PP10+ was 14.9% and 1.4%, respectively. In comparison, the prevalence of PP5+ was 22.6% and the prevalence of PP10+ was 9.8% among CPCSSN patients. When the two cohorts were categorized based on the co-occurrence of multimorbidity and polypharmacy, 14.3% of CLSA participants and 13.5% of CPCSSN patients were living with MM2+ and PP5+ (that is, both multimorbidity and polypharmacy). The most common conditions between the two cohorts were hypertension (CLSA: 36.9%; CPCSSN: 34.7%), obesity (CLSA: 29.7%; CPCSSN: 42.9%) and musculoskeletal problem (CLSA: 27.9%; CPCSSN: 38.7%). The most common medications between the two cohorts were HMG CoA reductase inhibitors (CLSA: 21.0%; CPCSSN: 12.5%), thyroid hormones (CLSA: 12.3%; CPCSSN: 6.0%) and proton pump inhibitors (CLSA: 11.7%; CPCSSN: 13.4%). The frequencies of all conditions and medications that were included in the operational definitions of multimorbidity and polypharmacy are presented in S3 Table.

**Table 1:** Characteristics and prevalence of multimorbidity and polypharmacy in CLSA and CPCSSN.

	CLSA (N = 30097)		CPCSSN (N = 597631)		p-value	
**Age Group**	n	%	n	%		
**45 to 64 Years**	17451	58.0	341732	57.2	0.04	
**65 to 85 Years**	12646	42.0	255899	42.8	0.08	
**Sex**						
**Female**	15320	50.9	328741	55.0	<0.01	
**Male**	14777	49.1	268890	45.0	<0.01	
**Mean Conditions (SD), Range**	2.6 (2.0)	0–15	1.9 (1.6)	0–14		
**Mean Medications (SD), Range**	2.2 (2.4)	0–21	3.0 (5.1)	0–88		
**Multimorbidity (MM)**						
**Two or More Conditions (MM2+)**	20060	66.7	310744	52.0	<0.01	
**Three or More Conditions (MM3+)**	13861	46.1	180536	30.2	<0.01	
**Polypharmacy (PP)**						
**Five or More Medications (PP5+)**	4492	14.9	135261	22.6	<0.01	
**Ten or More Medications (PP10+)**	405	1.4	58821	9.8	<0.01	
**Co-Occurrence of MM2+ and PP5+**						
**Neither MM2+ Nor PP5+**	9851	32.7	232362	38.9	<0.01	
**MM2+ Only**	15754	52.3	230008	38.5	<0.01	
**PP5+ Only**	186	0.6	54525	9.1	<0.01	
**Both MM2+ And PP5+**	4306	14.3	80736	13.5	0.1	
**Co-Occurrence of MM3+ and PP5+**						
**Neither MM3+ Nor PP5+**	15655	52.1	332521	55.6	<0.01	
**MM3+ Only**	9950	33.1	129849	21.7	<0.01	
**PP5+ Only**	581	1.9	84574	14.1	<0.01	
**Both MM3+ And PP5+**	3911	13.0	50687	8.5	<0.01	
**Most Frequent Conditions and Medications**						**Prevalence Difference (CLSA-CPCSSN)**
Hypertension	11096	**36.9**	207649	**34.7**	<0.01	2.1
Obesity	8933	**29.7**	256399	**42.9**	<0.01	-13.2
Osteoarthritis or rheumatoid arthritis	8434	**28.0**	82643	**13.8**	<0.01	14.2
Musculoskeletal problem	8384	**27.9**	231404	**38.7**	<0.01	-10.9
HMG CoA reductase inhibitors	6320	**21.0**	74593	**12.5**	<0.01	8.5
Anxiety or depression	6243	**20.7**	189064	**31.6**	<0.01	-10.9
Diabetes	5307	**17.6**	92954	**15.6**	<0.01	2.1
Chronic obstructive pulmonary disease or asthma	5093	**16.9**	69575	**11.6**	<0.01	5.3
Cancer	4636	**15.4**	146710	**24.5**	<0.01	-9.1
Thyroid problem	4376	**14.5**	58889	**9.9**	<0.01	4.7
Thyroid hormones	3706	**12.3**	36050	**6.0**	<0.01	6.3
Proton pump inhibitors	3523	**11.7**	80054	**13.4**	<0.01	-1.7
Heart failure	3503	**11.6**	10513	1.8	<0.01	9.9
Platelet aggregation inhibitors excl. heparin	3359	**11.2**	33444	**5.6**	<0.01	5.6
ACE inhibitors, plain	3030	**10.1**	48154	**8.1**	<0.01	2.0
Cardiovascular disease	2867	**9.5**	58744	**9.8**	<0.01	-0.3
Osteoporosis	2688	**8.9**	8971	1.5	<0.01	7.4
Urinary problem	2514	**8.4**	63918	**10.7**	<0.01	-2.3
Stomach problem	2275	**7.6**	31240	**5.2**	<0.01	2.3
Beta blocking agents, selective	2166	**7.2**	33447	**5.6**	<0.01	1.6
Angiotensin II receptor blockers (ARBs) and diuretics	2019	**6.7**	388	0.1	<0.01	6.6
Dihydropyridine derivatives	1784	**5.9**	31847	**5.3**	0.3	0.6
Other antidepressants	1760	**5.8**	48587	**8.1**	<0.01	-2.3
Thiazides, plain	1729	**5.7**	27759	4.6	0.04	1.1
Selective serotonin reuptake inhibitors	1570	**5.2**	55173	**9.2**	<0.01	-4.0
Anilides	1117	3.7	41022	**6.9**	<0.01	-3.2
Propionic acid derivatives	1050	3.5	56223	**9.4**	<0.01	-5.9
Benzodiazepine derivatives	986	3.3	59637	**10.0**	<0.01	-6.7
Adrenergics in combination with corticosteroids or other drugs, excl. anticholinergics	913	3.0	31903	**5.3**	<0.01	-2.3
Biguanides	901	3.0	31374	**5.2**	<0.01	-2.3
Selective beta-2-adrenoreceptor agonists	900	3.0	64945	**10.9**	<0.01	-7.9
Glucocorticoids	759	2.5	56990	**9.5**	<0.01	-7.0
Corticosteroids	718	2.4	87370	**14.6**	<0.01	-12.2
Colon problem	582	1.9	31081	**5.2**	<0.01	-3.3
Natural opium alkaloids	271	0.9	32766	**5.5**	<0.01	-4.6
Corticosteroids, potent (group III)	260	0.9	37299	**6.2**	<0.01	-5.4
Progestogens and estrogens, fixed combinations	84	0.3	40028	**6.7**	0.02	-6.4
Penicillins with extended spectrum	42	0.1	38606	**6.5**	0.09	-6.3
Fluoroquinolones	33	0.1	37953	**6.4**	0.14	-6.2
Macrolides	26	0.1	53389	**8.9**	0.12	-8.8

NB: CLSA participants were identified at baseline as of 2015 and CPCSSN patients were identified with any primary care encounter between 2014–2015; Only conditions and medications with an overall prevalence of at least 5.0% in either the CLSA or CPCSSN cohorts are presented, while the remaining conditions and medications are presented in S3 Table

### Middle-aged adults (45 to 64 years)

The results for the CLSA participants and CPCSSN patients aged 45 to 64 years are presented in Tables 2 and 3, respectively. Among CLSA participants, the overall prevalence of MM2+ was 57.7%, but female participants had a slightly higher prevalence of MM2+ as compared to males (61.7% and 53.5%, respectively). The prevalence of PP5+ was 10.0% and 9.1% among female and male CLSA participants aged 45 to 64 years, respectively. The highest proportion of CLSA participants were living with only MM2+ when categorized based on the co-occurrence of multimorbidity and polypharmacy (MM2+ and PP5+). The most frequent conditions among these CLSA participants were obesity, hypertension, musculoskeletal problem, anxiety or depression and osteoarthritis or rheumatoid arthritis. However, female CLSA participants had a higher prevalence of anxiety or depression (28.5%) as compared to male CLSA participants (18.1%) in this age group. There was more variation in the most frequent medications between females and males within this age group, such as a higher proportion of female participants taking thyroid hormones (14.5%) and higher proportion of male participants taking HMG CoA reductase inhibitors (17.7%).

**Table 2:** Prevalence and patterns of multimorbidity and polypharmacy in CLSA participants aged 45 to 64 years, overall and stratified by sex.

	All (n = 17451)		Females (n = 9014)			Males (n = 8437)		
**Mean Conditions (SD), Range**	2.2 (1.8)	0–15	2.4 (1.9)	0–15		2.0 (1.7)	0–10	
**Mean Medications (SD), Range**	1.6 (2.1)	0–20	1.7 (2.1)	0–19		1.5 (2.1)	0–20	
**Multimorbidity (MM)**								
**Two or More Conditions (MM2+)**	10075	57.7	5561	61.7		4514	53.5	
**Three or More Conditions (MM3+)**	6303	36.1	3596	39.9		2707	32.1	
**Polypharmacy (PP)**								
**Five or More Medications (PP5+)**	1670	9.6	903	10.0		767	9.1	
**Ten or More Medications (PP10+)**	156	0.9	84	0.9		72	0.9	
**Co-Occurrence of MM2+ and PP5+**								
**Neither MM2+ Nor PP5+**	7304	41.9	3417	37.9		3887	46.1	
**MM2+ Only**	8477	48.6	4694	52.1		3783	44.8	
**PP5+ Only**	72	0.4	36	0.4		36	0.4	
**Both MM2+ And PP5+**	1598	9.2	867	9.6		731	8.7	
**Co-Occurrence of MM3+ and PP5+**								
**Neither MM3+ Nor PP5+**	10898	62.5	5293	58.7		5605	66.4	
**MM3+ Only**	4883	28.0	2818	31.3		2065	24.5	
**PP5+ Only**	250	1.4	125	1.4		125	1.5	
**Both MM3+ And PP5+**	1420	8.1	778	8.6		642	7.6	
**Most Frequent Conditions**								
Obesity	5321	30.5	Obesity	2634	29.2	Obesity	2687	31.8
Hypertension	4856	27.8	Anxiety or depression	2569	28.5	Hypertension	2592	30.7
Musculoskeletal problem	4712	27.0	Musculoskeletal problem	2367	26.3	Musculoskeletal problem	2345	27.8
Anxiety or depression	4092	23.4	Osteoarthritis or rheumatoid arthritis	2276	25.2	Anxiety or depression	1523	18.1
Osteoarthritis or rheumatoid arthritis	3677	21.1	Hypertension	2264	25.1	Osteoarthritis or rheumatoid arthritis	1401	16.6
**Most Frequent Medications**								
HMG CoA reductase inhibitors	2433	13.9	Thyroid hormones	1309	14.5	HMG CoA reductase inhibitors	1496	17.7
Thyroid hormones	1665	9.5	HMG CoA reductase inhibitors	937	10.4	Platelet aggregation inhibitors excl. heparin	804	9.5
Proton pump inhibitors	1569	9.0	Proton pump inhibitors	885	9.8	ACE inhibitors, plain	749	8.9
Platelet aggregation inhibitors excl. heparin	1183	6.8	Other antidepressants	771	8.6	Proton pump inhibitors	684	8.1
ACE inhibitors, plain	1172	6.7	Natural and semisynthetic estrogens, plain	740	8.2	Beta blocking agents, selective	397	4.7

**Table 3:** Prevalence and patterns of multimorbidity and polypharmacy in CPCSSN patients aged 45 to 64 years, overall and stratified by sex.

	All (n = 341732)		Females (n = 189409)			Males (n = 152323)		
**Mean Conditions (SD), Range**	1.6 (1.4)	0–14	1.6 (1.5)	0–11		1.5 (1.3)	0–14	
**Mean Medications (SD), Range**	2.4 (4.2)	0–80	2.6 (4.5)	0–80		2.1 (3.8)	0–65	
**Multimorbidity (MM)**								
**Two or More Conditions (MM2+)**	150483	44.0	87511	46.2		62972	41.3	
**Three or More Conditions (MM3+)**	75306	22.0	45771	24.2		29535	19.4	
**Polypharmacy (PP)**								
**Five or More Medications (PP5+)**	61755	18.1	37743	19.9		24012	15.7	
**Ten or More Medications (PP10+)**	22374	6.5	14596	7.7		7778	5.1	
**Co-Occurrence of MM2+ and PP5+**								
**Neither MM2+ Nor PP5+**	163311	47.8	84879	44.8		78432	51.5	
**MM2+ Only**	116655	34.1	66787	35.3		49879	32.8	
**PP5+ Only**	27938	8.2	17019	9.0		10919	7.2	
**Both MM2+ And PP5+**	33817	9.9	20724	10.9		13093	8.6	
**Co-Occurrence of MM3+ and PP5+**								
**Neither MM3+ Nor PP5+**	223673	65.5	117829	62.2		105844	69.5	
**MM3+ Only**	56304	16.5	33837	17.9		22467	14.8	
**PP5+ Only**	42753	12.5	25809	13.6		16944	11.1	
**Both MM3+ And PP5+**	19002	5.5	11934	6.3		7068	4.6	
**Most Frequent Conditions**								
Obesity	101250	29.6	Obesity	54922	29.0	Obesity	46328	30.4
Musculoskeletal problem	92101	27.0	Musculoskeletal problem	52537	27.7	Musculoskeletal problem	39564	26.0
Anxiety or depression	68848	20.1	Anxiety or depression	44764	23.6	Hypertension	33999	22.3
Hypertension	66117	19.3	Cancer	33514	17.7	Anxiety or depression	24084	15.8
Cancer	50316	14.7	Hypertension	32118	17.0	Cancer	16802	11.0
**Most Frequent Medications**								
Corticosteroids	31851	9.3	Corticosteroids	19563	10.3	HMG CoA reductase inhibitors	13016	8.5
Proton pump inhibitors	27460	8.0	Proton pump inhibitors	15693	8.3	Corticosteroids	12288	8.1
Propionic acid derivatives	24123	7.1	Propionic acid derivatives	14496	7.7	Proton pump inhibitors	11767	7.7
Benzodiazepine derivatives	21709	6.4	Benzodiazepine derivatives	14443	7.6	Propionic acid derivatives	9627	6.3
Selective beta-2-adrenoreceptor agonists	21433	6.3	Selective beta-2-adrenoreceptor agonists	13430	7.1	ACE inhibitors, plain	8136	5.3

Among CPCSSN patients aged 45 to 64 years, the prevalence of MM2+ and PP5+ was 44.0% and 18.1%, respectively. The proportion of CPCSSN patients in this age group who were categorized as having both MM2+ and PP5+ was 9.9%, which was comparable to 9.2% of CLSA participants in the same age group who were categorized as living with both MM2+ and PP5+. These similar proportions were despite the fact that the CLSA middle-aged participants had a higher prevalence of MM2+ (CLSA: 57.7%; CPCSSN: 44.0%) and the CPCSSN middle-aged patients had a higher prevalence of PP5+ (CLSA: 9.6%; CPCSSN: 18.1%). The most frequent conditions among CPCSSN patients were similar to the CLSA participants in the same age group, including obesity, hypertension, musculoskeletal problem and anxiety or depression. The most common medication among CPCSSN patients aged 45 to 64 years was corticosteroids (10.3%) for females and HMG CoA reductase inhibitors (8.5%) for males.

### Older adults (65 to 85 years)

The results for the CLSA participants and CPCSSN patients aged 65 to 85 years are presented in Tables 4 and 5, respectively. In this age group, the prevalence of MM2+ was 79.0% and the prevalence of PP5+ was 22.3% among CLSA participants, while the prevalence of MM2+ was 62.6% and the prevalence of PP5+ was 28.7% among CPCSSN patients. Approximately 20.0% of CLSA participants and CPCSSN patients in this age group were identified as living with both MM2+ and PP5+ (CLSA: 21.4%; CPCSSN: 18.3%). The most common conditions among the CLSA participants (overall and stratified by sex) were hypertension (49.3%) and osteoarthritis or rheumatoid arthritis (37.6%). In comparison, the most common conditions among CPCSSN patients (overall and stratified by sex) were hypertension (43.5%) and obesity (30.4%). The most common medication for the older adults in the CLSA and CPCSSN cohorts, including when stratified between females and males, was HMG CoA reductase inhibitors (CLSA: 30.7%; CPCSSN: 18.1%).

**Table 4:** Prevalence and patterns of multimorbidity and polypharmacy in CLSA participants aged 65 to 85 years, overall and stratified by sex.

	**All (n = 12646)**		**Females (n = 6306)**			**Males (n = 6340)**		
**Mean Conditions (SD), Range**	3.3 (2.1)	0–13	3.5 (2.1)	0–13		3.0 (2.0)	0–12	
**Mean Medications (SD), Range**	2.9 (2.5)	0–21	2.9 (2.5)	0–21		2.9 (2.5)	0–18	
**Multimorbidity (MM)**								
**Two or More Conditions (MM2+)**	9985	79.0	5220	82.8		4765	75.2	
**Three or More Conditions (MM3+)**	7558	59.8	4100	65.0		3458	54.5	
**Polypharmacy (PP)**								
**Five or More Medications (PP5+)**	2822	22.3	1391	22.1		1431	22.6	
**Ten or More Medications (PP10+)**	249	2.0	127	2.0		122	1.9	
**Co-Occurrence of MM2+ and PP5+**								
**Neither MM2+ Nor PP5+**	2547	20.1	1050	16.7		1497	23.6	
**MM2+ Only**	7277	57.5	3865	61.3		3412	53.8	
**PP5+ Only**	114	0.9	36	0.6		78	1.2	
**Both MM2+ And PP5+**	2708	21.4	1355	21.5		1353	21.3	
**Co-Occurrence of MM3+ and PP5+**								
**Neither MM3+ Nor PP5+**	4757	37.6	2089	33.1		2668	42.1	
**MM3+ Only**	5067	40.1	2826	44.8		2241	35.4	
**PP5+ Only**	331	2.6	117	1.9		214	3.4	
**Both MM3+ And PP5+**	2491	19.7	1274	20.2		1217	19.2	
**Most Frequent Conditions**								
Hypertension	6240	49.3	Hypertension	3106	49.3	Hypertension	3134	49.4
Osteoarthritis or rheumatoid arthritis	4757	37.6	Osteoarthritis or rheumatoid arthritis	2934	46.5	Osteoarthritis or rheumatoid arthritis	1823	28.8
Musculoskeletal problem	3672	29.0	Obesity	1890	30.0	Musculoskeletal problem	1810	28.5
Obesity	3612	28.6	Musculoskeletal problem	1862	29.5	Obesity	1722	27.2
Cancer	2939	23.2	Thyroid problem	1663	26.4	Cancer	1584	25.0
**Most Frequent Medications**								
HMG CoA reductase inhibitors	3887	30.7	HMG CoA reductase inhibitors	1520	24.1	HMG CoA reductase inhibitors	2367	37.3
Platelet aggregation inhibitors excl. heparin	2176	17.2	Thyroid hormones	1458	23.1	Platelet aggregation inhibitors excl. heparin	1352	21.3
Thyroid hormones	2041	16.1	Proton pump inhibitors	1078	17.1	ACE inhibitors, plain	1149	18.1
Proton pump inhibitors	1954	15.5	Platelet aggregation inhibitors excl. heparin	824	13.1	Beta blocking agents, selective	940	14.8
ACE inhibitors, plain	1858	14.7	ACE inhibitors, plain	709	11.2	Proton pump inhibitors	876	13.8

**Table 5:** Prevalence and patterns of multimorbidity and polypharmacy in CPCSSN patients aged 65 to 85 years, overall and stratified by sex.

	All (n = 255899)		Females (n = 139332)			Males (n = 116567)		
**Mean Conditions (SD), Range**	2.3 (1.8)	0–14	2.4 (1.8)	0–14		2.3 (1.7)	0–13	
**Mean Medications (SD), Range**	3.8 (6.0)	0–88	3.9 (6.2)	0–88		3.6 (5.7)	0–68	
**Multimorbidity (MM)**								
**Two or More Conditions (MM2+)**	160261	62.6	88115	63.2		72146	61.9	
**Three or More Conditions (MM3+)**	105230	41.1	58820	42.2		46410	39.8	
**Polypharmacy (PP)**								
**Five or More Medications (PP5+)**	73506	28.7	40871	29.3		32635	28.1	
**Ten or More Medications (PP10+)**	36447	14.2	20933	15.0		15514	13.4	
**Co-Occurrence of MM2+ and PP5+**								
**Neither MM2+ Nor PP5+**	69051	27.0	36521	26.2		32530	27.9	
**MM2+ Only**	113342	44.3	61940	44.5		51402	44.1	
**PP5+ Only**	26587	10.4	14696	10.5		11891	10.2	
**Both MM2+ And PP5+**	46919	18.3	26175	18.8		20744	17.8	
**Co-Occurrence of MM3+ and PP5+**								
**Neither MM3+ Nor PP5+**	108848	42.5	57547	41.3		51301	44.0	
**MM3+ Only**	73545	28.7	40914	29.4		32631	28.0	
**PP5+ Only**	41821	16.3	22965	16.5		18856	16.2	
**Both MM3+ And PP5+**	31685	12.4	17906	12.8		13779	11.8	
**Most Frequent Conditions**								
Hypertension	111233	43.5	Hypertension	60037	43.1	Hypertension	51196	43.9
Obesity	77802	30.4	Obesity	41374	29.7	Obesity	36428	31.3
Musculoskeletal problem	70426	27.5	Musculoskeletal problem	40531	29.1	Musculoskeletal problem	29895	25.6
Cancer	53935	21.1	Cancer	29334	21.1	Diabetes	26990	23.2
Diabetes	50452	19.7	Osteoarthritis or rheumatoid arthritis	28602	20.5	Cancer	24601	21.1
**Most Frequent Medications**								
HMG CoA reductase inhibitors	46350	18.1	HMG CoA reductase inhibitors	21312	15.3	HMG CoA reductase inhibitors	25038	21.5
Proton pump inhibitors	32242	12.6	Proton pump inhibitors	18682	13.4	ACE inhibitors, plain	15290	13.1
ACE inhibitors, plain	27494	10.7	Corticosteroids	14192	10.2	Proton pump inhibitors	13560	11.6
Corticosteroids	23861	9.3	Benzodiazepine derivatives	13671	9.8	Platelet aggregation inhibitors excl. heparin	12239	10.5
Platelet aggregation inhibitors excl. heparin	21169	8.3	Glucocorticoids	12394	8.9	Beta blocking agents, selective	11154	9.6

**Fig 1:**
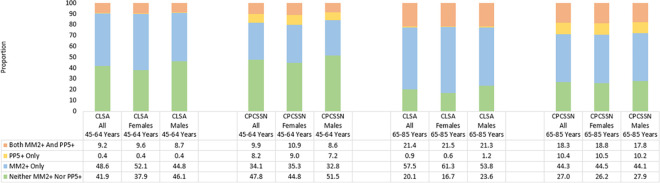
Prevalence of multimorbidity (two or more conditions) and polypharmacy (five or more medications) in CLSA and CPCSSN cohorts, stratified by age group and sex.

**Fig 2:**
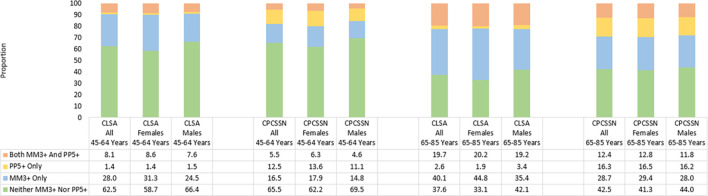
Prevalence of multimorbidity (three or more conditions) and polypharmacy (five or more medications) in CLSA and CPCSSN cohorts, stratified by age group and sex.

The comparative distributions of the CLSA and CPCSSN cohorts between the four mutually exclusive categories based on the co-occurrence of multimorbidity and polypharmacy (neither MM nor PP, MM only, PP only and both MM and PP), stratified by age group (45 to 64 years and 65 to 85 years) and by sex (female and male), are presented in Fig 1 (for MM2+ and PP5+) and Fig 2 (for MM3+ and PP5+).

## Discussion

### Summary of results

When defined as two or more conditions, the overall prevalence of multimorbidity was 66.7% in the CLSA cohort and 52.0% in the CPCSSN cohort. When defined as five or more medications, the overall prevalence of polypharmacy was 14.9% in the CLSA cohort and 22.6% in the CPCSSN cohort. Using these same two definitions, the co-occurrence of MM2+ and PP5+ was 14.3% among CLSA participants and 13.5% among CPCSSN patients. When stratified between age groups (45 to 64 years and 65 to 85 years), the co-occurrence of MM2+ and PP5+ was lower in middle-aged adults (CLSA: 9.2%; CPCSSN: 9.9%) as compared to older adults (CLSA: 21.4%; CPCSSN: 18.3%). As seen in Fig 1, the proportion of those with this co-occurrence is much smaller than those living with only multimorbidity. While polypharmacy without multimorbidity is rare, multimorbidity without polypharmacy is common. The prevalence of multimorbidity and polypharmacy was consistently higher among females as compared to males in both datasets and across both age groups. However, these differences between females and males were minor as the largest difference in prevalence estimates was observed among CLSA participants aged 65 to 85 years when reporting the prevalence of three or more conditions (females: 65.0%; males: 54.5%).

The differences in the prevalence of multimorbidity and polypharmacy between the CLSA and CPCSSN cohorts may be due to many factors including differences in the sample (community-dwelling vs primary care) or differences in the way that data were captured (self-reported vs diagnostic codes). Despite the differences in the data collection methods and the moderate differences in the overall prevalence of multimorbidity and polypharmacy between the CLSA and CPCSSN cohorts individually, there were still consistencies in the co-occurrence of multimorbidity and polypharmacy, which was found to be approximately one in five older adults in both cohorts. Individuals living with multimorbidity do not necessarily have polypharmacy, while those living with polypharmacy almost invariably have multimorbidity. Our results show that multimorbidity and polypharmacy are not interchangeable in understanding population health needs. Indeed, those living with both multimorbidity and polypharmacy likely represents an important group for focus in both the approach to clinical practice and health policy.

### Findings in context

Differences still exist between studies in the lists of individual conditions and medications that are used in the operational definitions of these concepts and this has an impact on resulting prevalence statistics [57]. As such, comparisons were made with studies that utilized the same cut-points for multimorbidity (two or more) and polypharmacy (five or more) as used in the current study. The prevalence of multimorbidity in the Canadian Chronic Disease Surveillance System (CCDSS) in 2015–2016 was found to be 32.4% among community-dwelling adults aged 35 years and older [80]. In the Canadian Community Health Survey (CCHS), the prevalence of multimorbidity in 2017–2018 was approximately 34.1% of community-dwelling adults aged 65 years and older [81]. Both of these estimates of multimorbidity were lower than the prevalence of multimorbidity in the current study. For the prevalence of polypharmacy, the Canadian Health Measures Survey (CHMS) in 2016–2017 found that 12.6% of community-dwelling adults aged 40 to 59 years and 32.1% of community-dwelling adults 60 to 79 years were taking five or more prescription medications [82], which were comparable to the prevalence of polypharmacy in the current study. The Canadian Institutes for Health Information (CIHI) in 2016 found that 65.7% of adults aged 65 years and older in Canada were prescribed five or more medication classes, which was substantially higher than the percentages reported in the current study as this administrative dataset included those living in long-term care settings [83].

In the context of other national health survey datasets, the CLSA cohort had a higher prevalence of multimorbidity and a lower prevalence of polypharmacy as compared to studies of the Longitudinal Aging Study Amsterdam (LASA) where multimorbidity prevalence was 45.3% and polypharmacy prevalence was 36.8% among participants aged 65 years and older [84]; the Irish Longitudinal Study on Ageing (TILDA) where multimorbidity prevalence was 59.3% and polypharmacy prevalence was 20.8% among participants aged 50 years and older [85]; and the Survey of Health, Ageing and Retirement in Europe (SHARE) where multimorbidity prevalence was 49.6% and polypharmacy prevalence was 22.3% among participants aged 50 years and older living in the eighteen countries that participated in wave 6 data collection [86]. In contrast, the prevalence of polypharmacy in the CLSA cohort was similar to that reported using the Brazilian Longitudinal Study of Adult Health (ELSA-Brasil), which was 11.7% among participants who were 35 to 74 years of age [87]. However, it is challenging to articulate if the differences in the prevalence estimates of multimorbidity and polypharmacy that are observed between these cohorts represent true differences or represent the impact of varying lists of conditions and medications.

In comparison with other large primary care datasets, the prevalence of both multimorbidity and polypharmacy in the CPCSSN cohort was comparable, but slightly lower than the prevalence reported by Sinnige et al. [88] using the NIVEL Primary Care Database (NIVEL-PCD) in the Netherlands. Amongst 45731 patients aged 55 years and older who received primary care from participating practices, the prevalence of multimorbidity was 58.0% and the prevalence of polypharmacy was 27.0% [88]. Another study in the Netherlands by van den Akker et al. [89] used Intego, a Flemish-Belgian general practice-based morbidity registration network at the Academic Centre of General Practice of the KU Leuven, which found a lower prevalence of multimorbidity (46.5%) and a higher prevalence of polypharmacy (40.7%) than the current study amongst primary care patients aged 50 years and older. In contrast, a study by O’Regan et al. [90] using EMR data in Ireland among patients aged 50 years and older who received primary care reported a lower prevalence of multimorbidity (37.6%), but a higher prevalence of polypharmacy (38.5%) than those estimates reported from the CPCSSN cohort. This study examined prevalence among 6603 patients from 68 general practices associated with the University of Limerick Education and Research Network for General Practice (ULEARN-GP) [90]. Interestingly, a higher prevalence of polypharmacy than multimorbidity is not common in other literature and was not seen in the results of the current study.

### Strengths and limitations

The strengths of this study include the application of a consistent definition and cut-point in identifying multimorbidity and polypharmacy across two national datasets. Although this required modification of a previously used definition of multimorbidity by Fortin et al. [59] as not all 20 conditions in the original multimorbidity definition were captured in the CLSA data, this consistent methodology supported the comparison between the two cohorts (including same age groups between samples). It was also important to present age- and sex-stratified analyses (gender was not captured in the CPCSSN data) to articulate differences between these groups as both multimorbidity and polypharmacy are known to be influenced by age, sex and gender [91, 92]. This study presents one of the first reports of the co-occurrence of multimorbidity and polypharmacy (in addition to reporting on the individual prevalence estimates) using two large and representative datasets in Canada, which can continue to be used for longitudinal surveillance of these two significant health states. In addition to facilitating international comparisons, large population-based datasets should play a key role in reporting on the changing occurrence of multimorbidity and polypharmacy over time, as well as contributing to the practice of pharmacovigilance [93].

The limitations of this study include the fact that multimorbidity and polypharmacy estimates were only stratified using age and sex categories due to the lack of more comprehensive information in the CPCSSN EMR database. The CLSA dataset contains a broad spectrum of extensive variables related to health and well-being (such as socioeconomic factors and health behaviours), but similar variables were not available in the CPCSSN dataset [73, 77]. Ideally, more comprehensive data should be entered and extracted from EMRs for clinical and research purposes. The medications data in the CPCSSN dataset were also included if there were no stop dates documented with the intent to capture “active” medications. However, this field may not be consistently documented by the primary care provider and the prescribed medications may have been stopped even if a stop date was not entered into the EMR. As well, the CLSA contains self-reported use of natural and non-prescribed products, which were not captured in the CPCSSN dataset, but can contribute to the complexity of a treatment regimen and should be explored in future research. Finally, although the data collection sites for both the CLSA and CPCSSN are located in many of the same cities in Canada, there is no current linkage to identify participants and patients who may be in both datasets, but there would be significant benefit of linking these two datasets for future research (including the validation between self-reported and clinically documented health conditions and current medications).

### Policy and practice implications

The presence of multimorbidity and polypharmacy can have substantial impact on the health status, health outcomes and health care use by individuals. This study emphasizes the need for a shift away from single disease clinical guidelines, toward clinical practice pathways that account for the co-occurrence of multiple conditions and treatments. Data from this study indicate that this is particularly important for individuals aged 65 years and older, as the proportion with co-occurrence was double that amongst middle-aged adults. The magnitude of benefits and side effects also shift with increasing age, as can individual preferences and priorities for treatment. A starting point for the shift away from single disease approaches would be to develop a completely different approach and separate pathways for this age group. This would support and guide discussions around the appropriate use of medications and other non-pharmacological interventions to facilitate effective management, as well as to reduce treatment burden [3, 4, 17]. While population level data cannot assess individual appropriateness of polypharmacy, multimorbidity does not have to result in substantial or problematic polypharmacy if clinicians are judicious in rational prescribing. This can be achieved through approaches to unwanted or unnecessary medications such as deprescribing, eliminating legacy prescribing (when medications that were appropriate at initiation for an intermediate term are not appropriately discontinued and subsequently lead to adverse outcomes) [18], using medications that can treat multiple co-existing conditions, reducing doses or discontinuing medications or combinations where the risks are too great [3, 94]. Health policy needs to take this broader view when considering resourcing, defining quality of care and related measurement and incentives. Practice and policy should continue to emphasize the value of patient-centered and team-focused approaches to providing comprehensive health care.

### Future research

This study did not examine any patient-important outcomes of concurrent multimorbidity and polypharmacy (such as quality of life, treatment burden or adverse events), but future research should continue to examine these outcomes and their occurrence in the context of independent and concurrent multimorbidity and polypharmacy. More nuanced information can come from assessing the impact, both separately and combined, as well as examining sub-analyses of patterns and combinations of morbidities, risk factors and related medications. Even further, research should continue to examine the impact of socioeconomic status and lifestyle behaviours, although these were not explored in the current study. While pharmaceutical management of risk factors can add to treatment burden, this is also a useful proxy marker for the severity of the condition, such as hypertension. Further analysis of related health service utilization and health system costs can provide useful information for policy and planning. Ideally, future research should leverage multiple sources of data to triangulate information from different perspectives, as we have in this study, which can provide insights into the potential for prevention and intervention at clinical and population levels.

## Conclusion

This study reported on the prevalence of the co-occurrence of multimorbidity and polypharmacy among middle-aged and older adults in two national datasets in Canada. Each dataset has strengths and limitations that can be leveraged in interpretation, and so combined the CLSA and CPCSSN datasets provide a more comprehensive assessment of these health states from both a population and primary care perspective. To inform health policy and clinical practice, future research and routine analyses should report on the co-occurrence of multimorbidity and polypharmacy. This insight can be used to further examine the impact and opportunities of interventions, evidence and clinical pathways that are tailored to individuals who are living with multimorbidity and polypharmacy in order to improve health outcomes over time.

## Supporting information

S1 Table List of conditions (ICD-9) for definition of multimorbidity. (PDF)

S2 Table List of medications (ATC Level 4) for definition of polypharmacy. (PDF)

S3 Table Prevalence of conditions and medications in CLSA and CPCSSN. (PDF)

## References

[1] World Health Organization. World Report on Aging and Health. Report. Luxembourg; 2015.

[2] PLOS Medicine Editors. Multimorbidity: addressing the next global pandemic. PLoS Med. 2023;20(4):e1004229. doi: 10.1371/journal.pmed.1004229 37014909 PMC10072481

[3] Daunt R, Curtin D, O’Mahony D. Polypharmacy stewardship: a novel approach to tackle a major public health crisis. Lancet Healthy Longev. 2023;4:e228–e235. doi: 10.1016/S2666-7568(23)00036-3 37030320

[4] Skou ST, Mair FS, Fortin M, Guthrie B, Nunes BP, Miranda JJ, et al. Multimorbidity. Nature Reviews Disease Primers. 2022;8:48. 10.1038/s41572-022-00376-4 PMC7613517 35835758

[5] Sirois C, Domingues NS, Laroche ML, Zongo A, Lunghi C, Guénette L, et al. Polypharmacy definitions for multimorbid older adults need stronger foundations to guide research, clinical practice and public health. Pharmacy (Basel). 2019;7(3):126. doi: 10.3390/pharmacy7030126 31470621 PMC6789889

[6] Aggarwal P, Woolford SJ, Patel HP. Multi-morbidity and polypharmacy in older people: challenges and opportunities for clinical practice. Geriatrics (Basel). 2020;5(4):85. doi: 10.3390/geriatrics5040085 33126470 PMC7709573

[7] Mercer SW, Salisbury C, Fortin M, eds. ABC of Multimorbidity. Oxford: John Wiley & Sons, 2014.

[8] Sinnott C, Bradley CP. Multimorbidity or polypharmacy: two sides of the same coin? J Comorb. 2015;5:29–31. doi: 10.15256/joc.2015.5.51 29090158 PMC5636041

[9] Doos L, Roberts EO, Corp N, Kadam UT. Multi-drug therapy in chronic condition multimorbidity: a systematic review. Fam Pract. 2014;31(6):654–663. doi: 10.1093/fampra/cmu056 25192902 PMC5942538

[10] Wise J. Polypharmacy: a necessary evil. BMJ. 2013;347:f7033. doi: 10.1136/bmj.f7033 24286985

[11] Muth C, Glasziou PP. Guideline recommended treatments in complex patients with multimorbidity. BMJ. 2015;351:h5145. doi: 10.1136/bmj.h5145 26431846

[12] Wallace E, Salisbury C, Guthrie B, Lewis C, Fahey T, Smith SM. Managing patients with multimorbidity in primary care. BMJ. 2015;350:h176. doi: 10.1136/bmj.h176 25646760

[13] Guthrie B, Payne K, Alderson P, McMurdo ME, Mercer SW. Adapting clinical guidelines to take account of multimorbidity. BMJ. 2012;345:e6341. doi: 10.1136/bmj.e6341 23036829

[14] Fortin M, Dionne J, Pinho G, Gignac J, Almirall J, Lapointe L. Randomized controlled trials: do they have external validity for patients with multiple comorbidities? Ann Fam Med. 2006;4(2):104–108. doi: 10.1370/afm.516 16569712 PMC1467012

[15] Boyd CM, Darer J, Boult C, Fried LP, Boult L, Wu AW. Clinical practice guidelines and quality of care for older patients with multiple comorbid diseases: implications for pay for performance. J Am Med Assoc. 2005;294:716–724. doi: 10.1001/jama.294.6.716 16091574

[16] Tinetti ME, Bogardus ST Jr, Agostini JV. Potential pitfalls of disease specific guidelines for patients with multiple conditions. N Engl J Med. 2004;351:2870–2874. doi: 10.1056/NEJMsb042458 15625341

[17] Onder G, Vetrano DL, Palmer K, Trevisan C, Amato L, Berti F, et al. Italian guidelines on management of persons with multimorbidity and polypharmacy. Aging Clin Exp Res. 2022;34(5):989–996. doi: 10.1007/s40520-022-02094-z 35249211 PMC9135855

[18] Mangin D, Lawson J, Cuppage J, Shaw E, Ivanyi K, Davis A, et al. Legacy drug prescribing patterns and associations in primary care: cohort study in the MUSIC Practice Based Research Network. Ann Fam Med. 2018;16(6):515–520. 30420366 10.1370/afm.2315 PMC6231929

[19] Mangin D, Stephen G, Bismah V, Risdon C. Making patient values visible in healthcare: a systematic review of tools to assess patient treatment priorities and preferences in the context of multimorbidity. BMJ Open. 2016:6:e010903. doi: 10.1136/bmjopen-2015-010903 27288377 PMC4908882

[20] Rosbach M, Andersen JS. Patient-experienced burden of treatment in patients with multimorbidity—a systematic review of qualitative data. PLoS One. 2017;12(6):e0179916–e0179934. doi: 10.1371/journal.pone.0179916 28644877 PMC5482482

[21] Morris JE, Roderick PJ, Harris S, Yao G, Crowe S, Phillips D, et al. Treatment burden for patients with multimorbidity: cross-sectional study with exploration of a single-item measure. Br J Gen Pract. 2021;71(706):e381–e390. doi: 10.3399/BJGP.2020.0883 33875419 PMC8074644

[22] Academy of Medical Sciences. Multimorbidity: a priority for global health research. United Kingdom; 2018.

[23] Delara M, Murray L, Jafari B, Bahji A, Goodarzi Z, Kirkham J, et al. Prevalence and factors associated with polypharmacy: a systematic review and meta-analysis. BMC Geriatr. 2022;22(1):601. doi: 10.1186/s12877-022-03279-x 35854209 PMC9297624

[24] Hsu HF, Chen KM, Belcastro F, Chen YF. Polypharmacy and pattern of medication use in community-dwelling older adults: a systematic review. J Clin Nurs. 2021;30(7–8):918–928. doi: 10.1111/jocn.15595 33325067

[25] Johnston MC, Crilly M, Black C, Prescott GJ, Mercer SW. Defining and measuring multimorbidity: a systematic review of systematic reviews. Eur J Public Health. 2019;29(1):182–189. doi: 10.1093/eurpub/cky098 29878097

[26] Marengoni A, Angleman S, Melis R, Mangialasche F, Karp A, Garmen A, et al. Aging with multimorbidity: a systematic review of the literature. Ageing Res Rev. 2011;10(4):430–439. doi: 10.1016/j.arr.2011.03.003 21402176

[27] Nguyen H, Manolova G, Daskalopoulou C, Vitoratou S, Prince M, Prina AM. Prevalence of multimorbidity in community settings: a systematic review and meta-analysis of observational studies. J Comorb. 2019;9:2235042X19870934. doi: 10.1177/2235042X19870934 31489279 PMC6710708

[28] Wastesson JW, Morin L, Tan ECK, Johnell K. An update on the clinical consequences of polypharmacy in older adults: a narrative review. Expert Opin Drug Saf. 2018;17(12):1185–1196. doi: 10.1080/14740338.2018.1546841 30540223

[29] Willadsen TG, Bebe A, Køster-Rasmussen R, Jarbøl DE, Guassora AD, Waldorff FB, et al. The role of diseases, risk factors and symptoms in the definition of multimorbidity—a systematic review. Scand J Prim Health Care. 2016;34(2):112–121. doi: 10.3109/02813432.2016.1153242 26954365 PMC4977932

[30] Chua YP, Xie Y, Lee PSS, Lee ES. Definitions and prevalence of multimorbidity in large database studies: a scoping review. Int J Environ Res Public Health. 2021;18(4):1673. doi: 10.3390/ijerph18041673 33572441 PMC7916224

[31] Davies LE, Spiers G, Kingston A, Todd A, Adamson J, Hanratty B. Adverse outcomes of polypharmacy in older people: systematic review of reviews. J Am Med Dir Assoc. 2020;21(2):181–187. doi: 10.1016/j.jamda.2019.10.022 31926797

[32] Fortin M, Lapointe L, Hudon C, Vanasse A, Ntetu AL, Maltais D. Multimorbidity and quality of life in primary care: a systematic review. Health Qual Life Outcomes. 2004;2:51. doi: 10.1186/1477-7525-2-51 15380021 PMC526383

[33] Fried TR, O’Leary J, Towle V, Goldstein MK, Trentalange M, Martin DK. Health outcomes associated with polypharmacy in community-dwelling older adults: a systematic review. J Am Geriatr Soc. 2014;62(12):2261–2272. doi: 10.1111/jgs.13153 25516023 PMC4270076

[34] Huntley AL, Johnson R, Purdy S, Valderas JM, Salisbury C. Measures of multimorbidity and morbidity burden for use in primary care and community settings: a systematic review and guide. Ann Fam Med. 2012;10(2):134–141. doi: 10.1370/afm.1363 22412005 PMC3315139

[35] Makovski TT, Schmitz S, Zeegers MP, Stranges S, van den Akker M. Multimorbidity and quality of life: systematic literature review and meta-analysis. Ageing Res Rev. 2019;53:100903. doi: 10.1016/j.arr.2019.04.005 31048032

[36] Masnoon N, Shakib S, Kalisch Ellett L, Caughey GE. What is polypharmacy? A systematic review of definitions. BMC Geriatrics. 2017;17(1):230. doi: 10.1186/s12877-017-0621-2 29017448 PMC5635569

[37] Pazan F, Wehling M. Polypharmacy in older adults: a narrative review of definitions, epidemiology and consequences. Eur Geriatr Med. 2021;12(3):443–452. doi: 10.1007/s41999-021-00479-3 33694123 PMC8149355

[38] Xu X, Mishra GD, Jones M. Evidence on multimorbidity from definition to intervention: an overview of systematic reviews. Ageing Res Rev. 2017;37:53–68. doi: 10.1016/j.arr.2017.05.003 28511964

[39] Gnjidic D, Pearson SA, Hilmer SN. Optimizing the impact of drugs on symptom burden in older people with multimorbidity at the end of life. JAMA Intern Med. 2014;174(4):636–637. doi: 10.1001/jamainternmed.2013.12875 24711180

[40] Guthrie B, Makubate B, Hernandez-Santiago V, Dreischulte T. The rising tide of polypharmacy and drug-drug interactions: population database analysis 1995–2010. BMC Med. 2015;13:74. doi: 10.1186/s12916-015-0322-7 25889849 PMC4417329

[41] Mangin D, Heath I. Multimorbidity and quaternary prevention (P4). Rev Bras Med Fam Comunidade. 2015;10(35):1–5.

[42] Noël PH, Frueh BC, Larme AC, Pugh JA. Collaborative care needs and preferences of primary care patients with multimorbidity. Heal Expect. 2005;8:54–63. doi: 10.1111/j.1369-7625.2004.00312.x 15713171 PMC5060269

[43] Boyd CM, Fortin M. Future of multimorbidity research: how should understanding of multimorbidity inform health system design? Public Health Rev. 2010;32(2):451–474.

[44] Bayliss EA, Ellis JL, Steiner JF. Barriers to self-management and quality-of-life outcomes in seniors with multimorbidities. Ann Fam Med. 2007;5(5):395–402. doi: 10.1370/afm.722 17893380 PMC2000313

[45] Bayliss EA, Steiner JF, Fernald DH, Crane LA, Main DS. Descriptions of barriers to self-care by persons with comorbid chronic diseases. Ann Fam Med. 2003;1(1):15–21. doi: 10.1370/afm.4 15043175 PMC1466563

[46] Agborsangaya CB, Lau D, Lahtinen M, Cooke T, Johnson JA. Health-related quality of life and healthcare utilization in multimorbidity: results of a cross-sectional survey. Qual Life Res. 2013;22:791–799. doi: 10.1007/s11136-012-0214-7 22684529

[47] Fortin M, Soubhi H, Hudon C, Bayliss EA, van den Akker M. Multimorbidity’s many challenges: time to focus on the needs of this vulnerable and growing population. BMJ. 2007;334:1016–1017. 17510108 10.1136/bmj.39201.463819.2C PMC1871747

[48] Chang AY, Bryazka D, Dieleman JL. Estimating health spending associated with chronic multimorbidity in 2018: an observational study among adults in the United States. PLoS Med. 2023;20(4):e1004205. doi: 10.1371/journal.pmed.1004205 37014826 PMC10072449

[49] Clark CM, Shaver AL, Aurelio LA, Feuerstein S, Wahler RG Jr, Daly CJ, et al. Potentially inappropriate medications are associated with increased healthcare utilization and costs. J Am Geriatr Soc. 2020;68(11):2542–2550. doi: 10.1111/jgs.16743 32757494 PMC8235922

[50] Hartmann J, Hehner S, Hemmrich K, Kors B, Mohlmann T. Providing better care at lower cost for multimorbid patients. Heal Int. 2011;11:38–47.

[51] Vogeli C, Shields AE, Lee TA, Gibson TB, Marder WD, Weiss KB, et al. Multiple chronic conditions: prevalence, health consequences, and implications for quality, care management, and costs. J Gen Intern Med. 2007;22:391–395. doi: 10.1007/s11606-007-0322-1 18026807 PMC2150598

[52] Rapoport J, Jacobs P, Bell NR, Klarenbach S. Refining the measurement of the economic burden of chronic diseases in Canada. Chronic Dis Can. 2004;25(1):13–21. 15298484

[53] Gill A, Kuluski K, Jaakkimainen L, Naganathan G, Upshur R, Wodchis WP. “Where do we go from here?” Health system frustrations expressed by patients with multimorbidity, their caregivers and family physicians. Healthc Policy. 2014;9(4):73–89. 24973485 PMC4749886

[54] Gustafsson M, Kristensson J, Holst G, Willman A, Bohman D. Case managers for older persons with multi-morbidity and their everyday work—a focused ethnography. BMC Health Serv Res. 2013;13:496–511. doi: 10.1186/1472-6963-13-496 24279695 PMC3893533

[55] Salisbury C, Johnson L, Purdy S, Valderas JM, Montgomery AA. Epidemiology and impact of multimorbidity in primary care: a retrospective cohort study. Br J Gen Pract. 2011;61(582):e12. doi: 10.3399/bjgp11X548929 21401985 PMC3020068

[56] Ali MU, Sherifali D, Fitzpatrick-Lewis D, Kenny M, Lamarche L, Raina P, et al. Interventions to address polypharmacy in older adults living with multimorbidity: review of reviews. Canadian Family Physician. 2022; 68(7):e215–e226. doi: 10.46747/cfp.6807e215 35831093 PMC9842141

[57] Nicholson K, Liu W, Fitzpatrick D, Hardacre KA, Roberts S, Salerno J, et al. Prevalence of multimorbidity and polypharmacy among adults and older adults: a systematic review. Lancet Healthy Longev. 2024:S2666-7568(24)00007-2. doi: 10.1016/S2666-7568(24)00007-2 38452787

[58] Liu H, Zhao Y, Qiao L, Yang C, Yang Y, Zhang T, et al. Consistency between self-reported disease diagnosis and clinical assessment and under-reporting for chronic conditions: data from a community-based study in Xi’an, China. Front Public Health. 2024;12:1296939. doi: 10.3389/fpubh.2024.1296939 38292908 PMC10825002

[59] Fortin M, Haggerty J, Sanche S, Almirall J. Self-reported versus health administrative data: implications for assessing chronic illness burden in populations. A cross-sectional study. CMAJ Open. 2017;5(3):e729–e733. doi: 10.9778/cmajo.20170029 28947426 PMC5621946

[60] Muggah E, Graves E, Bennett C, Manuel DG. Ascertainment of chronic diseases using population health data: a comparison of health administrative data and patient self-report. BMC Public Health. 2013;13:16. doi: 10.1186/1471-2458-13-16 23302258 PMC3557162

[61] Matsumoto M, Harada S, Iida M, Kato S, Sata M, Hirata A, et al. Validity assessment of self-reported medication use for hypertension, diabetes, and dyslipidemia in a pharmacoepidemiologic study by comparison with health insurance claims. J Epidemiol. 2021;31(9):495–502. doi: 10.2188/jea.JE20200089 33361656 PMC8328856

[62] Hafferty JD, Campbell AI, Navrady LB, Adams MJ, MacIntyre D, Lawrie SM, et al. Self-reported medication use validated through record linkage to national prescribing data. J Clin Epidemiol. 2018;94:132–142. doi: 10.1016/j.jclinepi.2017.10.013 29097340 PMC5808931

[63] Okura Y, Urban LH, Mahoney DW, Jacobsen SJ, Rodeheffer RJ. Agreement between self-report questionnaires and medical record data was substantial for diabetes, hypertension, myocardial infarction and stroke but not for heart failure. J Clin Epidemiol. 2004;57(10):1096–1103. doi: 10.1016/j.jclinepi.2004.04.005 15528061

[64] Garies S, Cummings M, Forst B, McBrien K, Soos B, Taylor M, et al. Achieving quality primary care data: a description of the Canadian Primary Care Sentinel Surveillance Network data capture, extraction, and processing in Alberta. Int J Popul Data Sci. 2019;4(2):1132. doi: 10.23889/ijpds.v4i2.1132 34095540 PMC8142949

[65] Singer A, Kroeker AL, Yakubovich S, Duarte R, Dufault B, Katz A. Data quality in electronic medical records in Manitoba: Do problem lists reflect chronic disease as defined by prescriptions? Can Fam Physician. 2017;63(5):382–389. 28500199 PMC5429058

[66] Muller S. Electronic medical records: the way forward for primary care research? Fam Pract. 2014;31(2):127–129. doi: 10.1093/fampra/cmu009 24627543 PMC3969524

[67] Terry AL, Chevendra V, Thind A, Stewart M, Marshall JN, Cejic S. Using your electronic medical record for research: a primer for avoiding pitfalls. Fam Pract. 2010;27(1):121–126. doi: 10.1093/fampra/cmp068 19828572

[68] Canadian Longitudinal Study on Aging. Canadian Longitudinal Study on Aging Data Collection. 2024. Available from: https://www.clsa-elcv.ca/.

[69] Raina P, Wolfson C, Kirkland S, Griffith LE, Balion C, Cossette B, et al. Cohort Profile: the Canadian Longitudinal Study on Aging (CLSA). Int J Epidemiol. 2019;48(6):1752–1753j. doi: 10.1093/ije/dyz173 31633757 PMC6929533

[70] Raina PS, Wolfson C, Kirkland SA, Griffith LE, Oremus M, Patterson C, et al. The Canadian Longitudinal Study on Aging (CLSA). Can J Aging. 2009;28(3):221–229. doi: 10.1017/S0714980809990055 19860977

[71] Cossette B, Griffith L, Emond PD, Mangin D, Moss L, Boyko J, et al. Drug and natural health product data collection and curation in the Canadian Longitudinal Study on Aging (CLSA). Can J Aging. 2024;1–7. 10.1017/S0714980823000806 38268103

[72] Canadian Primary Care Sentinel Surveillance Network. Canadian Primary Care Sentinel Surveillance Network. 2024. Available from: http://cpcssn.ca/.

[73] Garies S, Birtwhistle R, Drummond N, Queenan J, Williamson T. Data Resource Profile: National electronic medical record data from the Canadian Primary Care Sentinel Surveillance Network (CPCSSN). Int J Epidemiol. 2017;46(4):1091–1092f. doi: 10.1093/ije/dyw248 28338877

[74] Birtwhistle R. Canadian Primary Care Sentinel Surveillance Network: a developing resource for family medicine and public health. Can Fam Physician. 2011;57:1219–1220. 21998241 PMC3192094

[75] Birtwhistle R, Keshavjee K, Lambert-Lanning A, Godwin M, Greiver M, Manca D, et al. Building a pan-Canadian primary care sentinel surveillance network: initial development and moving forward. J Am Board Fam Med. 2009;22(4):412–22. doi: 10.3122/jabfm.2009.04.090081 19587256

[76] Morkem R, Salman A, Herman C, Shah R, Barber D. CPCSSN data and information quality: an opportunity for enhancing Canadian primary care data. Canada; 2023.

[77] Queenan JA, Williamson T, Khan S, Drummond N, Garies S, Morkem R, et al. Representativeness of patients and providers in the Canadian Primary Care Sentinel Surveillance Network: a cross-sectional study. CMAJ Open. 2016;4(1):e28–e32. doi: 10.9778/cmajo.20140128 27331051 PMC4866925

[78] Fortin M, Almirall J, Nicholson K. Development of a research tool to document self-reported chronic conditions in primary care. J Comorbidity. 2017;7(1):117–123. doi: 10.15256/joc.2017.7.122 29354597 PMC5772378

[79] StataCorp. 2023. Stata Statistical Software: Release 17. College Station, TX: StataCorp LLC.

[80] Public Health Agency of Canada. Canadian Chronic Disease Surveillance System (CCDSS). 2024. Available from: https://health-infobase.canada.ca/ccdss/data-tool/

[81] Public Health Agency of Canada. Aging and chronic diseases: a profile of Canadian seniors. 2020. Available from: https://www.canada.ca/content/dam/hc-sc/documents/services/publications/diseases-and-conditions/aging-chronic-diseases/canadian-seniors-report_2021-eng.pdf

[82] Harris DA, Guo Y, Nakhla N, Tadrous M, Hogan DB, Hennessy D, et al. Prevalence of prescription and non-prescription polypharmacy by frailty and sex among middle-aged and older Canadians. Health Rep. 2022;33(6):3–16. doi: 10.25318/82-003-x202200600001-eng 35876612

[83] Canadian Institute for Health Information. Drug Use Among Seniors in Canada, 2016. Canada; 2018.

[84] de Breij S, Rijnhart JJM, Schuster NA, Rietman ML, Peters MJL, Hoogendijk EO. Explaining the association between frailty and mortality in older adults: the mediating role of lifestyle, social, psychological, cognitive, and physical factors. Prev Med Rep. 2021;24:101589. doi: 10.1016/j.pmedr.2021.101589 34976648 PMC8683887

[85] Jacob L, Shin JI, Kostev K, Haro JM, López-Sánchez GF, Smith L, et al. Prospective association between multimorbidity and falls and its mediators: findings from the Irish Longitudinal Study on Ageing. J Clin Med. 2022;11(15):4470. doi: 10.3390/jcm11154470 35956086 PMC9370027

[86] Makovski TT, Le Coroller G, Putrik P, Choi YH, Zeegers MP, Stranges S, et al. Role of clinical, functional and social factors in the association between multimorbidity and quality of life: findings from the Survey of Health, Ageing and Retirement in Europe (SHARE). PLoS One. 2020;15(10):e0240024. doi: 10.1371/journal.pone.0240024 33079931 PMC7575102

[87] Silva IR, Gonçalves LG, Chor D, Fonseca MJMD, Mengue SS, Acurcio FA, et al. Polypharmacy, socioeconomic indicators and number of diseases: results from ELSA-Brasil. Rev Bras Epidemiol. 2020;23:e200077. doi: 10.1590/1980-549720200077 32638852

[88] Sinnige J, Braspenning JC, Schellevis FG, Hek K, Stirbu I, Westert GP, et al. Inter-practice variation in polypharmacy prevalence amongst older patients in primary care. Pharmacoepidemiol Drug Saf. 2016;25(9):1033–1041. doi: 10.1002/pds.4016 27133740

[89] van den Akker M, Vaes B, Goderis G, Van Pottelbergh G, De Burghgraeve T, Henrard S. Trends in multimorbidity and polypharmacy in the Flemish-Belgian population between 2000 and 2015. PLoS One. 2019;14(2):e0212046. doi: 10.1371/journal.pone.0212046 30753214 PMC6372187

[90] O’Regan A, O’Doherty J, O’Connor R, Cullen W, Niranjan V, Glynn L, et al. How do multi-morbidity and polypharmacy affect general practice attendance and referral rates? A retrospective analysis of consultations. PLoS One. 2022;17(2):e0263258. doi: 10.1371/journal.pone.0263258 35113926 PMC8812985

[91] Mangin D. A Primary Care Perspective on Prescribing for Women. Medicines for Women. Ed. Harrison-Woolrych M. 2015; Springer International Publishing.

[92] Phillips SP. Including gender in public health research. Public Health Rep. 2011;126 Suppl:16–21. doi: 10.1177/00333549111260S304 21836732 PMC3150124

[93] Williamson T, Lévesque L, Morkem R, Birtwhistle R. CPCSSN’s role in improving pharmacovigilance. Can Fam Physician. 2014;60(7):678–680. 25022644 PMC4096271

[94] Mangin D, Bahat G, Golomb BA, Mallery LH, Moorhouse P, Onder G, et al. International Group for Reducing Inappropriate Medication Use & Polypharmacy (IGRIMUP): position statement and 10 recommendations for action. Drugs Aging. 2018;35(7):575–587. 30006810 10.1007/s40266-018-0554-2 PMC6061397

