# Supplementary material for: The co-occurrence of multimorbidity and polypharmacy among middle-aged and older adults in Canada: A cross-sectional study using the Canadian Longitudinal Study on Aging (CLSA) and the Canadian Primary Care Sentinel Surveillance Network (CPCSSN)
Source: PLoS One. 2025 Jan 15;20(1):e0312873. doi: 10.1371/journal.pone.0312873 (PMC11734935; doi:10.1371/journal.pone.0312873)
Supplement: S1 Table — (PDF) [file pone.0312873.s001.pdf]

S1 Table: List of conditions (ICD-9) for definition of multimorbidity

Anxiety or depression (296, 296.2, 296.21, 296.22, 296.23, 296.24, 296.25, 296.26, 296.3, 296.31, 296.32, 296.33, 296.34, 296.35, 296.36, 300, 300.01, 300.02, 300.09)

Cancer (140 - 239)

Cardiovascular disease (412, 413, 413.1, 413.2, 440 - 449, 427, 427.3, 427.31, 427.32)

Chronic obstructive pulmonary disease or asthma (491, 491.1, 491.2, 491.21, 491.22, 491.8, 491.9, 492, 492.8, 493, 493.01, 493.02, 493.1, 493.11, 493.12, 493.2, 493.21, 493.22, 493.8, 493.81, 493.82, 493.9, 493.91, 493.92)

Colon problem (555, 555.1, 555.2, 555.9, 556, 556.4, 556.5, 556.6, 556.8, 556.9, 564, 564.1)

Dementia (290, 290.1, 290.11, 290.12, 290.13, 290.2, 290.21, 290.3, 290.4, 294, 294.1, 294.2)

Diabetes (250, 250.01, 250.02, 250.03, 250.1, 250.11, 250.12, 250.13, 250.2, 250.21, 250.22, 250.23, 250.3, 250.31, 250.32, 250.33, 250.4, 250.41, 250.42, 250.43, 250.5, 250.51, 250.52, 250.53, 250.6, 250.61, 250.62, 250.63, 250.7, 250.71, 250.72, 250.73, 250.8, 250.81, 250.82, 250.83, 250.9, 250.91, 250.92, 250.93)

Heart failure (428, 394, 394.1, 394.2, 395, 395.1, 395.2, 395.9)

Hypertension (401 - 405, 401, 401.1, 401.9, 405, 405.01, 405.09, 405.1, 405.11, 405.19, 405.9, 405.91, 405.99)

Kidney disease or failure (585, 585.1, 585.2, 585.3, 585.4, 585.5, 585.6, 585.9)

Musculoskeletal problem (723, 723.1, 724, 724.1, 724.2, 724.3, 724.4, 724.5, 725, 726, 726.1, 726.2, 726.3, 726.31, 726.32, 726.33, 726.39, 726.4, 726.5, 726.6, 726.61, 726.62, 726.63, 726.64, 726.65, 726.69, 726.7, 726.71, 726.72, 726.73, 726.79, 726.9, 726.91, 727, 727.01, 727.03, 727.04, 727.05, 727.06, 727.09, 727.2, 727.3, 729, 729.1, 729.2, 729.4, 729.5)

Obesity (278, 278.01, BMI >= 30)

Osteoarthritis or rheumatoid arthritis (714, 714.1, 714.2, 714.3, 715, 715.1, 715.2, 715.3, 715.8, 715.9)

Osteoporosis (733, 733.01, 733.02, 733.03, 733.09)

Stomach problem (530, 530.81, 531, 531.4, 531.41, 531.5, 531.51, 531.6, 531.61, 531.7, 531.71, 531.9, 531.91)

Stroke or transient ischemic attack (434, 434.01, 434.1, 434.11, 434.9, 434.91, 435, 435.1, 435.2, 435.3, 435.8, 435.9)

Thyroid problem (240 - 246, 240, 241, 242, 243, 244, 245, 246)

Urinary problem (593, 593.3, 593.4, 593.5, 593.7, 593.71, 593.72, 593.73, 593.8, 593.82, 593.89, 593.9, 595, 595.1, 595.2, 595.9, 597, 597.8, 597.81, 597.82, 600, 601, 601.1, 601.3, 601.8, 601.9, 602, 602.1, 602.2, 602.3, 602.8, 602.9)
