# Supplementary material for: The co-occurrence of multimorbidity and polypharmacy among middle-aged and older adults in Canada: A cross-sectional study using the Canadian Longitudinal Study on Aging (CLSA) and the Canadian Primary Care Sentinel Surveillance Network (CPCSSN)
Source: PLoS One. 2025 Jan 15;20(1):e0312873. doi: 10.1371/journal.pone.0312873 (PMC11734935; doi:10.1371/journal.pone.0312873)
Supplement: S2 Table — (PDF) [file pone.0312873.s002.pdf]

S2 Table: List of medications (ATC Level 4) for definition of polypharmacy

2-amino-1-phenylethanol derivatives (C04AA)
3-oxoandrosten (4) derivatives (G03BA)
5-androstanon (3) derivatives (G03BB)
ACE inhibitors and calcium channel blockers (C09BB)
ACE inhibitors and diuretics (C09BA)
ACE inhibitors, other combinations (C09BX)
ACE inhibitors, plain (C09AA)
Acetic acid derivatives and related substances (M01AB)
Acid preparations (A09AB)
Acidifiers (G04BA)
Acridine derivatives (D08AA)
ACTH (H01AA)
Actinomycines (L01DA)
Adamantane derivatives (N04BB)
Adrenergic and dopaminergic agents (C01CA)
Adrenergics and other drugs for obstructive airway diseases (R03CK)
Adrenergics in combination with anticholinergics incl. triple combinations with corticosteroids (R03AL)
Adrenergics in combination with corticosteroids or other drugs, excl. anticholinergics (R03AK)
Agents for dermatitis, excluding corticosteroids (D11AH)
Aldehydes and derivatives (N05CC)
Aldose reductase inhibitors (A10XA)
Aldosterone antagonists (C03DA)
Alkaloids, excl. rauwolfia (C02KA)
Alkaloids, excl. rauwolfia, in combination with diuretics (C02LK)
Alkyl sulfonates (L01AB)
Allergen extracts (V01AA)
Alpha and beta blocking agents (C07AG)
Alpha and beta blocking agents and other diuretics (C07CG)
Alpha and beta blocking agents and thiazides (C07BG)

Alpha- and beta-adrenoreceptor agonists (R03AA)
Alpha- and beta-adrenoreceptor agonists (R03CA)
Alpha glucosidase inhibitors (A10BF)
Alpha-adrenoreceptor antagonists (C02CA)
Alpha-adrenoreceptor antagonists (G04CA)
Alpha-adrenoreceptor antagonists and diuretics (C02LE)
Aluminium agents (D08AB)
Aluminium compounds (A02AB)
Amides (N01BB)
Amino acids (B02AA)
Amino acids (B05XB)
Amino acids and derivatives (A16AA)
Amino acids, incl. combinations with polypeptides (V06DD)
Amino acids/carbohydrates/minerals/vitamins, combinations (V06DE)
Aminoalkyl ethers (R06AA)
Aminoquinolines (P01BA)
Aminosalicylic acid and derivatives (J04AA)
Aminosalicylic acid and similar agents (A07EC)
Amphenicols (J01BA)
Analgesics and anesthetics (S02DA)
Anaplastic lymphoma kinase (ALK) inhibitors (L01ED)
Androgen, progestogen and estrogen in combination (G03EB)
Androgens and estrogens (G03EA)
Androgens and female sex hormones in combination with other drugs (G03EK)
Androgens for topical use (D11AE)
Androstan derivatives (A14AA)
Anesthetics for topical use (D04AB)
Anesthetics, local (R02AD)
Angiotensin II receptor blockers (ARBs) and calcium channel blockers (C09DB)
Angiotensin II receptor blockers (ARBs) and diuretics (C09DA)

Angiotensin II receptor blockers (ARBs), other combinations (C09DX)
Angiotensin II receptor blockers (ARBs), plain (C09CA)
Anilides (N02BE)
Antacids with antiflatulents (A02AF)
Antacids with antispasmodics (A02AG)
Antacids with sodium bicarbonate (A02AH)
Antacids, other combinations (A02AX)
Anthracyclines and related substances (L01DB)
Anthrax vaccines (J07AC)
Antiallergic agents, excl. corticosteroids (A07EB)
Antiallergic agents, excl. corticosteroids (R01AC)
Antiallergic agents, excl. corticosteroids (R03BC)
Anti-androgens (L02BB)
Antiandrogens and estrogens (G03HB)
Antiandrogens, plain (G03HA)
Antiarrhythmics, class Ia (C01BA)
Antiarrhythmics, class Ib (C01BB)
Antiarrhythmics, class Ic (C01BC)
Antiarrhythmics, class III (C01BD)
Antibiotics (A07AA)
Antibiotics (C05AB)
Antibiotics (D01AA)
Antibiotics (G01AA)
Antibiotics (J02AA)
Antibiotics (J04AB)
Antibiotics (R02AB)
Antibiotics (S01AA)
Antibiotics and corticosteroids (G01BA)
Anticholinergics (R03BB)
Anticholinergics (S01FA)

Anticholinesterases (N06DA)
Anticholinesterases (N07AA)
Anticorticosteroids (H02CA)
Antidepressants in combination with psycholeptics (N06CA)
Antidiarrheal microorganisms (A07FA)
Antidotes (V03AB)
Anti-estrogens (L02BA)
Antifungals for systemic use (D01BA)
Anti-gonadotropin-releasing hormones (H01CC)
Antigonadotropins and similar agents (G03XA)
Antihidrotics (D11AA)
Antihistamines for topical use (D04AA)
Antihypertensives for pulmonary arterial hypertension (C02KX)
Antiinfectives (B05CA)
Antiinfectives (S02AA)
Antiinfectives (S03AA)
Antiinfectives and antiseptics for local oral treatment (A01AB)
Antiinfectives for treatment of acne (D10AF)
Antiinflammatory agents, non-steroids (S01BC)
Antiinflammatory agents, non-steroids and antiinfectives in combination (S01CC)
Antiinflammatory preparations, non-steroids for topical use (M02AA)
Antiinflammatory products for vaginal administration (G02CC)
Antiinflammatory/antirheumatic agents in combination with corticosteroids (M01BA)
Antimony compounds (P01CB)
Antineovascularisation agents (S01LA)
Antipropulsives (A07DA)
Antiseptics (R02AA)
Antiseptics and corticosteroids (G01BD)
Antispasmodics in combination with other drugs (A03ED)
Antispasmodics, psycholeptics and analgesics in combination (A03EA)

Antivertigo preparations (N07CA)
Antivirals (D06BB)
Antivirals (S01AD)
Antivirals for treatment of HCV infections (J05AP)
Antivirals for treatment of HIV infections, combinations (J05AR)
Antracen derivatives (D05AC)
Aromatase inhibitors (L02BG)
Arsenic compounds (G01AB)
Arsenic compounds (P01AR)
Arsenic compounds (P01CD)
Artemisinin and derivatives, combinations (P01BF)
Artemisinin and derivatives, plain (P01BE)
Aryloxyacetic acid derivatives (C03CC)
Ascorbic acid (vitamin C), combinations (A11GB)
Ascorbic acid (vitamin C), plain (A11GA)
Avermectines (P02CF)
Azaspirodecanedione derivatives (N05BE)
Bacterial and viral vaccines, combined (J07CA)
Barbiturates and derivatives (N03AA)
Barbiturates in combination with other drugs (N01AG)
Barbiturates, combinations (N05CB)
Barbiturates, plain (N01AF)
Barbiturates, plain (N05CA)
Barium sulfate containing X-ray contrast media (V08BA)
Bcr-abl tyrosine kinase inhibitors (L01EA)
Belladonna alkaloids, semisynthetic, quaternary ammonium compounds (A03BB)
Belladonna alkaloids, tertiary amines (A03BA)
Belladonna and derivatives in combination with analgesics (A03DB)
Belladonna and derivatives in combination with psycholeptics (A03CB)
Benzamides (N05AL)

Benzimidazole derivatives (P02CA)
Benzodiazepine derivatives (N03AE)
Benzodiazepine derivatives (N05BA)
Benzodiazepine derivatives (N05CD)
Benzodiazepine related drugs (N05CF)
Benzomorphan derivatives (N02AD)
Benzothiazepine derivatives (C08DB)
Beta blocking agents (S01ED)
Beta blocking agents and calcium channel blockers (C07FB)
Beta blocking agents, non-selective (C07AA)
Beta blocking agents, non-selective, and other diuretics (C07CA)
Beta blocking agents, non-selective, and thiazides (C07BA)
Beta blocking agents, non-selective, and vasodilators (C07EA)
Beta blocking agents, non-selective, thiazides and other diuretics (C07DA)
Beta blocking agents, other combinations (C07FX)
Beta blocking agents, selective (C07AB)
Beta blocking agents, selective, and other diuretics (C07CB)
Beta blocking agents, selective, and thiazides (C07BB)
Beta blocking agents, selective, and vasodilators (C07EB)
Beta blocking agents, selective, thiazides and other diuretics (C07DB)
Beta-lactamase inhibitors (J01CG)
Beta-lactamase resistant penicillins (J01CF)
Beta-lactamase sensitive penicillins (J01CE)
Biguanides (A10BA)
Biguanides (P01BB)
Biguanides and amidines (D08AC)
Bile acid sequestrants (C10AC)
Bile acids and derivatives (A05AA)
Bioflavonoids (C05CA)
Bismuth preparations (A07BB)

Bisphosphonates (M05BA)
Bisphosphonates, combinations (M05BB)
Bisquaternary ammonium compounds (C02BC)
Blood coagulation factors (B02BD)
Blood substitutes and plasma protein fractions (B05AA)
Blood tests, auxiliary products (V07AD)
Blood transfusion, auxiliary products (V07AC)
Bone morphogenetic proteins (M05BC)
Boric acid products (D08AD)
B-raf serine-threonine kinase (BRAF) inhibitors (L01EC)
Brucellosis vaccines (J07AD)
Bruton's tyrosine kinase (BTK) inhibitors (L01EL)
Bulk-forming laxatives (A06AC)
Butylpyrazolidines (M01AA)
Butyrophenone derivatives (N05AD)
Calcineurin inhibitors (L04AD)
Calcitonin gene-related peptide (CGRP) antagonists (N02CD)
Calcitonin preparations (H05BA)
Calcium (A12AA)
Calcium channel blockers and diuretics (C08GA)
Calcium compounds (A02AC)
Calcium, combinations with vitamin D and/or other drugs (A12AX)
Capsaicin and similar agents (M02AB)
Carbamates (N05BC)
Carbamic acid esters (M03BA)
Carbamide products (D02AE)
Carbapenems (J01DH)
Carbohydrates (V06DC)
Carbohydrates/proteins/minerals/vitamins, combinations (V06DA)
Carbonic anhydrase inhibitors (S01EC)

Carboxamide derivatives (N03AF)
Caries prophylactic agents (A01AA)
Centrally acting antiobesity products (A08AA)
Centrally acting sympathomimetics (N06BA)
Charcoal preparations (A07BA)
Chemicals and reagents for analysis (V07AZ)
Chlorine containing products (P03AB)
Cholera vaccines (J07AE)
Choline derivatives (M03AB)
Choline esters (N07AB)
Cod-liver oil ointments (D03AA)
Colchicine derivatives (L01CC)
Colony stimulating factors (L03AA)
Colouring agents (S01JA)
Combinations and complexes of aluminium, calcium and magnesium compounds (A02AD)
Combinations for eradication of Helicobacter pylori (A02BD)
Combinations of adrenergics (R03AH)
Combinations of antibacterials (J01RA)
Combinations of antihistamines (R06AK)
Combinations of antineoplastic agents (L01XY)
Combinations of drugs for treatment of tuberculosis (J04AM)
Combinations of oral blood glucose lowering drugs (A10BD)
Combinations of penicillins, incl. beta-lactamase inhibitors (J01CR)
Combinations of sulfonamides and trimethoprim, incl. derivatives (J01EE)
Combinations of vitamins (A11JA)
Contact laxatives (A06AB)
Corticosteroid derivatives (N02CB)
Corticosteroids (C05AA)
Corticosteroids (R01AD)
Corticosteroids (S02BA)

Corticosteroids (S03BA)
Corticosteroids acting locally (A07EA)
Corticosteroids and antiinfectives in combination (S01CA)
Corticosteroids and antiinfectives in combination (S02CA)
Corticosteroids and antiinfectives in combination (S03CA)
Corticosteroids and mydriatics in combination (S01BB)
Corticosteroids for local oral treatment (A01AC)
Corticosteroids for systemic use, combinations (H02BX)
Corticosteroids, combinations for treatment of acne (D10AA)
Corticosteroids, moderately potent (group II) (D07AB)
Corticosteroids, moderately potent, combinations with antibiotics (D07CB)
Corticosteroids, moderately potent, combinations with antiseptics (D07BB)
Corticosteroids, moderately potent, other combinations (D07XB)
Corticosteroids, plain (S01BA)
Corticosteroids, potent (group III) (D07AC)
Corticosteroids, potent, combinations with antibiotics (D07CC)
Corticosteroids, potent, combinations with antiseptics (D07BC)
Corticosteroids, potent, other combinations (D07XC)
Corticosteroids, very potent (group IV) (D07AD)
Corticosteroids, very potent, combinations with antibiotics (D07CD)
Corticosteroids, very potent, combinations with antiseptics (D07BD)
Corticosteroids, very potent, other combinations (D07XD)
Corticosteroids, weak (group I) (D07AA)
Corticosteroids, weak, combinations with antibiotics (D07CA)
Corticosteroids, weak, combinations with antiseptics (D07BA)
Corticosteroids, weak, other combinations (D07XA)
Corticosteroids/antiinfectives/mydriatics in combination (S01CB)
Cosmetics (V07AT)
Coxibs (M01AH)
Curare alkaloids (M03AA)

Cyclic amines (J05AC)
Cyclin-dependent kinase (CDK) inhibitors (L01EF)
Dantrolene and derivatives (M03CA)
Detoxifying agents for antineoplastic treatment (V03AF)
Diaminopyrimidines (P01BD)
Diazepines, oxazepines, thiazepines and oxepines (N05AH)
Dibenzo-bicyclo-octadiene derivatives (N05BD)
Dichloroacetamide derivatives (P01AC)
Digitalis glycosides (C01AA)
Dihydropyridine derivatives (C08CA)
Dipeptidyl peptidase 4 (DPP-4) inhibitors (A10BH)
Diphenylbutylpiperidine derivatives (N05AG)
Diphenylmethane derivatives (N05BB)
Diphenylpropylamine derivatives (N02AC)
Diphtheria vaccines (J07AF)
Direct factor Xa inhibitors (B01AF)
Direct thrombin inhibitors (B01AE)
Dopa and dopa derivatives (N04BA)
Dopamine agonists (N04BC)
Drugs for embolisation (V03AM)
Drugs for treatment of hypercalcemia (V03AG)
Drugs for treatment of hyperkalemia and hyperphosphatemia (V03AE)
Drugs for treatment of hypoglycemia (V03AH)
Drugs for treatment of lepra (J04BA)
Drugs for urinary frequency and incontinence (G04BD)
Drugs used in alcohol dependence (N07BB)
Drugs used in erectile dysfunction (G04BE)
Drugs used in hereditary angioedema (B06AC)
Drugs used in nicotine dependence (N07BA)
Drugs used in opioid dependence (N07BC)

Electrolyte solutions (B05XA)
Emergency contraceptives (G03AD)
Encephalitis vaccines (J07BA)
Enemas (A06AG)
Enzyme and acid preparations, combinations (A09AC)
Enzyme preparations (A09AA)
Enzymes (A16AB)
Enzymes (B01AD)
Enzymes (B06AA)
Enzymes (C04AF)
Enzymes (M09AB)
Epidermal growth factor receptor (EGFR) tyrosine kinase inhibitors (L01EB)
Epoxides (L01AG)
Ergot alkaloids (C04AE)
Ergot alkaloids (G02AB)
Ergot alkaloids (N02CA)
Ergot alkaloids and oxytocin incl. analogues, in combination (G02AC)
Esters of aminobenzoic acid (N01BA)
Esters of benzoic acid (N01BC)
Estren derivatives (A14AB)
Estren derivatives (G03DC)
Estrogens (L02AA)
Estrogens, combinations with other drugs (G03CC)
Ethers (N01AA)
Ethers chemically close to antihistamines (N04AB)
Ethers of tropine or tropine derivatives (N04AC)
Ethers, chemically close to antihistamines (M03BC)
Ethylene imines (L01AC)
Expectorants (R05CA)
Fat/carbohydrates/proteins/minerals/vitamins, combinations (V06DB)

Fatty acid derivatives (N03AG)
Fenamates (M01AG)
Fibrates (C10AB)
Fibrinogen (B02BB)
First-generation cephalosporins (J01DB)
Fluoride (A12CD)
Fluoroquinolones (J01MA)
Fluoroquinolones (S01AE)
Folic acid analogues (L01BA)
Folic acid and derivatives (B03BB)
Fourth-generation cephalosporins (J01DE)
Gangliosides and ganglioside derivatives (N07XA)
Glucagon-like peptide-1 (GLP-1) analogues (A10BJ)
Glucocorticoids (H02AB)
Glucocorticoids (R03BA)
Glycogenolytic hormones (H04AA)
Glycopeptide antibacterials (J01XA)
Gold preparations (M01CB)
Gonadotropin releasing hormone analogues (L02AE)
Gonadotropin-releasing hormones (H01CA)
Gonadotropins (G03GA)
Guanidine derivatives (C02CC)
Guanidine derivatives (C02DG)
Guanidine derivatives and diuretics (C02LF)
H2-receptor antagonists (A02BA)
Haemophilus influenzae B vaccines (J07AG)
Halogenated hydrocarbons (N01AB)
Hedgehog pathway inhibitors (L01XJ)
Heme products (B06AB)
Hemodialytics, concentrates (B05ZA)

Hemofiltrates (B05ZB)
Heparin group (B01AB)
Heparins or heparinoids for topical use (C05BA)
Hepatitis vaccines (J07BC)
High-ceiling diuretics and potassium-sparing agents (C03EB)
Histone deacetylase (HDAC) inhibitors (L01XH)
HMG CoA reductase inhibitors (C10AA)
HMG CoA reductase inhibitors in combination with other lipid modifying agents (C10BA)
HMG CoA reductase inhibitors, other combinations (C10BX)
Human epidermal growth factor receptor 2 (HER2) tyrosine kinase inhibitors (L01EH)
Hydantoin derivatives (N03AB)
Hydrazides (J04AC)
Hydrazinophthalazine derivatives (C02DB)
Hydrazinophthalazine derivatives and diuretics (C02LG)
Hydroxyquinoline derivatives (P01AA)
Hypertonic solutions (B05DB)
Hypnotics and sedatives in combination, excl. barbiturates (N05CX)
Imidazole and triazole derivatives (D01AC)
Imidazole derivatives (A07AC)
Imidazole derivatives (G01AF)
Imidazole derivatives (J01XD)
Imidazole derivatives (J02AB)
Imidazole derivatives and corticosteroids (G01BF)
Imidazoline derivatives (C04AB)
Imidazoline receptor agonists (C02AC)
Imidazoline receptor agonists in combination with diuretics (C02LC)
Imidazothiazole derivatives (P02CE)
Immune sera (J06AA)
Immunoglobulins, normal human (J06BA)
Incontinence equipment (V07AN)

Indifferent preparations (S02DC)
Indium (111In) compounds (V09HB)
Indium (111In) compounds (V09IB)
Indole derivatives (N05AE)
Influenza vaccines (J07BB)
Insulins and analogues for inhalation (A10AF)
Insulins and analogues for injection, fast-acting (A10AB)
Insulins and analogues for injection, intermediate- or long-acting combined with fast-acting (A10AD)
Insulins and analogues for injection, intermediate-acting (A10AC)
Insulins and analogues for injection, long-acting (A10AE)
Integrase inhibitors (J05AJ)
Interferons (L03AB)
Interleukin inhibitors (L04AC)
Interleukins (L03AC)
Intermediate-acting sulfonamides (J01EC)
Intrauterine contraceptives (G02BA)
Intravaginal contraceptives (G02BB)
Iodine (123I) compounds (V09AB)
Iodine (125I) compounds (V09GB)
Iodine (131I) compounds (V09XA)
Iodine (131I) compounds (V10XA)
Iodine products (D08AG)
Iodine therapy; systemic (H03CA)
Iron bivalent, oral preparations (B03AA)
Iron chelating agents (V03AC)
Iron in combination with folic acid (B03AD)
Iron in other combinations (B03AE)
Iron trivalent, oral preparations (B03AB)
Iron, parenteral preparations; parenteral (B03AC)
Isotonic solutions (B05DA)

Janus-associated kinase (JAK) inhibitors (L01EJ)
Leukotriene receptor antagonists (R03DC)
Lincosamides (J01FF)
Liquid plasters (D02AD)
Lithium (N05AN)
Liver therapy (A05BA)
Local anesthetics (C05AD)
Local anesthetics (S01HA)
Local hemostatics (B02BC)
Long-acting sulfonamides (J01ED)
Low-ceiling diuretics and potassium-sparing agents (C03EA)
Low-energy diets (V06AA)
Lung surfactants (R07AA)
Macrolides (J01FA)
Magnesium (A12CC)
Magnesium compounds (A02AA)
Mammalian target of rapamycin (mTOR) kinase inhibitors (L01EG)
MAO inhibitors (C02KC)
MAO inhibitors and diuretics (C02LL)
Measles vaccines (J07BD)
Medical gases (V03AN)
Medicated dressings with antiinfectives (D09AA)
Medicated shampoos (D11AC)
Melatonin receptor agonists (N05CH)
Meningococcal vaccines (J07AH)
Mercurial diuretics (C03BC)
Mercurial products (D08AK)
Methanolquinolines (P01BC)
Methyldopa (C02AB)
Methyldopa and diuretics in combination (C02LB)

Methylhydrazines (L01XB)
Milk substitutes (V06DF)
Mineralocorticoids (H02AA)
Mitogen-activated protein kinase (MEK) inhibitors (L01EE)
Monoamine oxidase A inhibitors (N06AG)
Monoamine oxidase B inhibitors (N04BD)
Monoamine oxidase inhibitors, non-selective (N06AF)
Monobactams (J01DF)
Monoclonal antibodies (L01XC)
Morphinan derivatives (N02AF)
Mucolytics (R05CB)
Multivitamins with minerals (A11AA)
Multivitamins, other combinations (A11AB)
Multivitamins, plain (A11BA)
Mumps vaccines (J07BE)
Muscle relaxants (C05AE)
Natural and semisynthetic estrogens, plain (G03CA)
Natural opium alkaloids (N02AA)
Nerve depressants (V03AZ)
Neuraminidase inhibitors (J05AH)
Nicotinic acid and derivatives (C04AC)
Nicotinic acid and derivatives (C10AD)
Nitroferricyanide derivatives (C02DD)
Nitrofuran derivatives (D08AF)
Nitrofuran derivatives (J01XE)
Nitrofuran derivatives (P01CC)
Nitrogen mustard analogues (L01AA)
Nitroimidazole derivatives (P01AB)
Nitroimidazole derivatives (P01CA)
Nitrosoureas (L01AD)

Non-nucleoside reverse transcriptase inhibitors (J05AG)
Non-selective beta-adrenoreceptor agonists (R03AB)
Non-selective beta-adrenoreceptor agonists (R03CB)
Non-selective monoamine reuptake inhibitors (N06AA)
Non-watersoluble X-ray contrast media (V08AD)
Nucleoside and nucleotide reverse transcriptase inhibitors (J05AF)
Nucleosides and nucleotides excl. reverse transcriptase inhibitors (J05AB)
Nutrients without phenylalanine (V06CA)
Opioid anesthetics (N01AH)
Opioids in combination with antispasmodics (N02AG)
Opioids in combination with non-opioid analgesics (N02AJ)
Opium alkaloids and derivatives (R05DA)
Opium derivatives and expectorants (R05FA)
Oral rehydration salt formulations (A07CA)
Organic acids (G01AD)
Organic nitrates (C01DA)
Organophosphorous compounds (P02BB)
Oripavine derivatives (N02AE)
Osmotically acting laxatives (A06AD)
Other agents against amoebiasis and other protozoal diseases (P01AX)
Other agents against leishmaniasis and trypanosomiasis (P01CX)
Other agents for local oral treatment (A01AD)
Other agents for treatment of hemorrhoids and anal fissures for topical use (C05AX)
Other alkylating agents (L01AX)
Other aminoglycosides (J01GB)
Other analgesics and antipyretics (N02BG)
Other anterior pituitary lobe hormones and analogues (H01AX)
Other anti-acne preparations for systemic use (D10BX)
Other anti-acne preparations for topical use (D10AX)
Other antiallergics (S01GX)

Other antianemic preparations (B03XA)
Other antiarrhythmics, class I and III (C01BG)
Other antibacterials (J01XX)
Other antibiotics for topical use (D06AX)
Other anticestodals (P02DX)
Other anti-dementia drugs (N06DX)
Other antidepressants (N06AX)
Other antidiarrheals (A07XA)
Other antiemetics (A04AD)
Other antiepileptics (N03AX)
Other antifungals for topical use (D01AE)
Other antiglaucoma preparations (S01EX)
Other antigout preparations (M04AX)
Other antihistamines for systemic use (R06AX)
Other antihypertensives and diuretics (C02LX)
Other antiinfectives (S01AX)
Other antiinfectives and antiseptics (G01AX)
Other antiinflammatory and antirheumatic agents, non-steroids (M01AX)
Other antiinflammatory therapeutic radiopharmaceuticals (V10AX)
Other antiinflammatory/antirheumatic agents in combination with other drugs (M01BX)
Other antimalarials (P01BX)
Other antimigraine preparations (N02CX)
Other antimycotics for systemic use (J02AX)
Other antinematodals (P02CX)
Other antineoplastic agents (L01XX)
Other antiobesity drugs (A08AX)
Other anti-parathyroid agents (H05BX)
Other antiparkinson drugs (N04CX)
Other antipruritics (D04AX)
Other antipsoriatics for systemic use (D05BX)

Other antipsoriatics for topical use (D05AX)
Other antipsychotics (N05AX)
Other antiseptics and disinfectants (D08AX)
Other antispasmodics in combination with analgesics (A03DC)
Other antispasmodics in combination with psycholeptics (A03CC)
Other antithrombotic agents (B01AX)
Other antithyroid preparations (H03BX)
Other antitrematodal agents (P02BX)
Other antivirals (J05AX)
Other anxiolytics (N05BX)
Other bacterial vaccines (J07AX)
Other blood glucose lowering drugs, excl. insulins (A10BX)
Other blood products (B05AX)
Other capillary stabilizing agents (C05CX)
Other cardiac combination products (C01EX)
Other cardiac glycosides (C01AX)
Other cardiac preparations (C01EB)
Other cardiac stimulants (C01CX)
Other cardiovascular system diagnostic radiopharmaceuticals (V09GX)
Other central nervous system diagnostic radiopharmaceuticals (V09AX)
Other centrally acting agents (M03BX)
Other cephalosporins and penems (J01DI)
Other chemotherapeutics (D06BX)
Other cicatrizants (D03AX)
Other combinations of nutrients (V06DX)
Other cough suppressants (R05DB)
Other cough suppressants and expectorants (R05FB)
Other cytotoxic antibiotics (L01DC)
Other dermatologicals (D11AX)
Other diagnostic agents (V04CX)

Other diagnostic radiopharmaceuticals for inflammation and infection detection (V09HX)
Other diagnostic radiopharmaceuticals for tumour detection (V09IX)
Other dopaminergic agents (N04BX)
Other drugs affecting bone structure and mineralization (M05BX)
Other drugs for bile therapy (A05AX)
Other drugs for constipation (A06AX)
Other drugs for disorders of the musculo-skeletal system (M09AX)
Other drugs for functional gastrointestinal disorders (A03AX)
Other drugs for obstructive airway diseases, inhalants (R03BX)
Other drugs for peptic ulcer and gastro-oesophageal reflux disease (GORD) (A02BX)
Other drugs for treatment of tuberculosis (J04AK)
Other drugs used in benign prostatic hypertrophy (G04CX)
Other ectoparasiticides, incl. scabicides (P03AX)
Other emollients and protectives (D02AX)
Other estrogens (G03CX)
Other general anesthetics (N01AX)
Other gynecologicals (G02CX)
Other hematological agents (B06AX)
Other hepatic and reticulo endothelial system diagnostic radiopharmaceuticals (V09DX)
Other high-ceiling diuretics (C03CX)
Other hormone antagonists and related agents (L02BX)
Other hormones (L02AX)
Other hypnotics and sedatives (N05CM)
Other i.v. solution additives (B05XX)
Other immunoglobulins (J06BC)
Other immunostimulants (L03AX)
Other immunosuppressants (L04AX)
Other insecticides and repellents (P03BX)
Other intestinal adsorbents (A07BC)
Other intestinal antiinfectives (A07AX)

Other irrigating solutions (B05CX)
Other lipid modifying agents (C10AX)
Other local anesthetics (N01BX)
Other low-ceiling diuretics (C03BX)
Other magnetic resonance imaging contrast media (V08CX)
Other mineral products (A12CX)
Other muscle relaxants, peripherally acting agents (M03AX)
Other nasal preparations (R01AX)
Other nervous system drugs (N07XX)
Other non-selective calcium channel blockers (C08EX)
Other non-therapeutic auxiliary products (V07AY)
Other ophthalmological diagnostic agents (S01JX)
Other ophthalmologicals (S01XA)
Other opioids (N02AX)
Other parasympathomimetics (N07AX)
Other peripheral vasodilators (C04AX)
Other plain vitamin preparations (A11HA)
Other plant alkaloids and natural products (L01CX)
Other potassium-sparing agents (C03DB)
Other protein kinase inhibitors (L01EX)
Other psychostimulants and nootropics (N06BX)
Other quaternary ammonium compounds (M03AC)
Other quinolones (J01MB)
Other renal system diagnostic radiopharmaceuticals (V09CX)
Other respiratory system diagnostic radiopharmaceuticals (V09EX)
Other respiratory system products (R07AX)
Other sclerosing agents (C05BX)
Other selective calcium channel blockers with mainly vascular effects (C08CX)
Other sex hormones and modulators of the genital system (G03XX)
Other specific antirheumatic agents (M01CX)

Other surgical aids (S01KX)
Other systemic drugs for obstructive airway diseases (R03DX)
Other systemic hemostatics (B02BX)
Other therapeutic products (V03AX)
Other throat preparations (R02AX)
Other topical products for joint and muscular pain (M02AX)
Other urologicals (G04BX)
Other uterotonics (G02AX)
Other vasodilators used in cardiac diseases (C01DX)
Other viral vaccines (J07BX)
Ovulation stimulants, synthetic (G03GB)
Oxazol, thiazine, and triazine derivatives (M03BB)
Oxazolidine derivatives (N03AC)
Oxicams (M01AC)
Oxytocin and analogues (H01BB)
Papaverine and derivatives (A03AD)
Papillomavirus vaccines (J07BM)
Paramagnetic contrast media (V08CA)
Parasympathomimetics (S01EB)
Parathyroid hormones and analogues (H05AA)
Penicillamine and similar agents (M01CC)
Penicillins with extended spectrum (J01CA)
Perchlorates (H03BC)
Peripheral opioid receptor antagonists (A06AH)
Peripherally acting antiobesity products (A08AB)
Peroxides (D10AE)
Pertussis vaccines (J07AJ)
Phenol and derivatives (D08AE)
Phenothiazine derivatives (R06AD)
Phenothiazines with aliphatic side-chain (N05AA)

Phenothiazines with piperazine structure (N05AB)
Phenothiazines with piperidine structure (N05AC)
Phenylalkylamine derivatives (C08DA)
Phenylalkylamine derivatives (C08EA)
Phenylpiperidine derivatives (N02AB)
Phosphatidylinositol-3-kinase (Pi3K) inhibitors (L01EM)
Phosphodiesterase inhibitors (C01CE)
Phosphonic acid derivatives (J05AD)
Piperazine and derivatives (P02CB)
Piperazine derivatives (R06AE)
Piperidinedione derivatives (N05CE)
Plague vaccines (J07AK)
Plasters (V07AA)
Platelet aggregation inhibitors excl. heparin (B01AC)
Platinum compounds (L01XA)
Pneumococcal vaccines (J07AL)
Podophyllotoxin derivatives (L01CB)
Poliomyelitis vaccines (J07BF)
Poly (ADP-ribose) polymerase (PARP) inhibitors (L01XK)
Polymyxins (J01XB)
Potassium (A12BA)
Pregnadien derivatives (G03DB)
Pregnen (4) derivatives (G03DA)
Preparations containing sulfur (D10AB)
Preparations for biliary tract therapy (A05AB)
Preparations increasing uric acid excretion (M04AB)
Preparations inhibiting uric acid production (M04AA)
Preparations with no effect on uric acid metabolism (M04AC)
Preparations with salicylic acid derivatives (M02AC)
Progesterone receptor modulators (G03XB)

Progestogens (G03AC)
Progestogens (L02AB)
Progestogens and estrogens, fixed combinations (G03AA)
Progestogens and estrogens, fixed combinations (G03FA)
Progestogens and estrogens, sequential preparations (G03AB)
Progestogens and estrogens, sequential preparations (G03FB)
Prolactine inhibitors (G02CB)
Propionic acid derivatives (M01AE)
Propulsives (A03FA)
Prostaglandin analogues (S01EE)
Prostaglandins (A02BB)
Prostaglandins (C01EA)
Prostaglandins (G02AD)
Protease inhibitors (J05AE)
Proteasome inhibitors (L01XG)
Protectives against UV-radiation for systemic use (D02BB)
Protectives against UV-radiation for topical use (D02BA)
Proteinase inhibitors (B02AB)
Proteolytic enzymes (D03BA)
Proton pump inhibitors (A02BC)
Psoralens for systemic use (D05BA)
Psoralens for topical use (D05AD)
Psychostimulants in combination with psycholeptics (N06CB)
Purine analogues (L01BB)
Purine derivatives (C04AD)
Pyrazolone derivatives (C03CD)
Pyrazolones (N02BB)
Pyrethrines (P03BA)
Pyrethrines, incl. synthetic compounds (P03AC)
Pyrimidine analogues (L01BC)

Pyrimidine derivatives (C02DC)
Quaternary ammonium compounds (D08AJ)
Quinine and derivatives (M09AA)
Quinoline derivatives (D08AH)
Quinoline derivatives (G01AC)
Quinoline derivatives and corticosteroids (G01BC)
Quinoline derivatives and related substances (P02BA)
Quinolines (M01CA)
Quinolone vasodilators (C01DB)
Rabies vaccines (J07BG)
Rauwolfia alkaloids (C02AA)
Rauwolfia alkaloids and diuretics in combination (C02LA)
Renin-inhibitors (C09XA)
Respiratory stimulants (R07AB)
Retinoids for cancer treatment (L01XF)
Retinoids for topical use in acne (D10AD)
Retinoids for treatment of acne (D10BA)
Retinoids for treatment of psoriasis (D05BB)
Rota virus diarrhea vaccines (J07BH)
Rubella vaccines (J07BJ)
Salicylic acid and derivatives (N02BA)
Salicylic acid derivatives (P02DA)
Salicylic acid preparations (D02AF)
Salt solutions (B05CB)
Scilla glycosides (C01AB)
Sclerosing agents for local injection (C05BB)
Secondary and tertiary amines (C02BB)
Second-generation cephalosporins (J01DC)
Selective beta-2-adrenoreceptor agonists (R03AC)
Selective beta-2-adrenoreceptor agonists (R03CC)

Selective estrogen receptor modulators (G03XC)
Selective immunosuppressants (L04AA)
Selective serotonin (5HT1) agonists (N02CC)
Selective serotonin reuptake inhibitors (N06AB)
Selenium (A12CE)
Sensitivity tests, discs and tablets (V07AR)
Sensitizers used in photodynamic/radiation therapy (L01XD)
Serotonin (5HT3) antagonists (A04AA)
Serotonin antagonists (C02KD)
Serotonin antagonists and diuretics (C02LN)
Serotonin receptor antagonists (A03AE)
Short-acting sulfonamides (J01EB)
Silicone products (D02AA)
Silver compounds (D08AL)
Sodium (A12CA)
Sodium-glucose co-transporter 2 (SGLT2) inhibitors (A10BK)
Soft paraffin and fat products (D02AC)
Soft paraffin dressings (D09AX)
Softeners, emollients (A06AA)
Solutions affecting the electrolyte balance (B05BB)
Solutions for parenteral nutrition (B05BA)
Solutions producing osmotic diuresis (B05BC)
Solvents and diluting agents, incl. irrigating solutions (V07AB)
Somatostatin and analogues (H01CB)
Somatropin and somatropin agonists (H01AC)
Specific immunoglobulins (J06BB)
Steroid antibacterials (J01XC)
Stomi equipment (V07AS)
Streptogramins (J01FG)
Streptomycins (J01GA)

Strophanthus glycosides (C01AC)
Substituted alkylamines (R06AB)
Substituted ethylene diamines (R06AC)
Succinimide derivatives (N03AD)
Sulfonamides (A07AB)
Sulfonamides (D06BA)
Sulfonamides (G01AE)
Sulfonamides (heterocyclic) (A10BC)
Sulfonamides (S01AB)
Sulfonamides and corticosteroids (G01BE)
Sulfonamides and potassium in combination (C03BB)
Sulfonamides and potassium in combination (C03CB)
Sulfonamides, combinations with other drugs (C03BK)
Sulfonamides, plain (C03BA)
Sulfonamides, plain (C03CA)
Sulfonium derivatives (C02BA)
Sulfonylureas (A10BB)
Sulfur containing products (P03AA)
Sulfur-containing imidazole derivatives (H03BB)
Superparamagnetic contrast media (V08CB)
Sympathomimetics (R01BA)
Sympathomimetics excl. antiglaucoma preparations (S01FB)
Sympathomimetics in glaucoma therapy (S01EA)
Sympathomimetics used as decongestants (S01GA)
Sympathomimetics, combinations excl. corticosteroids (R01AB)
Sympathomimetics, labour repressants (G02CA)
Sympathomimetics, plain (R01AA)
Synthetic anticholinergic agents in combination with analgesics (A03DA)
Synthetic anticholinergic agents in combination with psycholeptics (A03CA)
Synthetic anticholinergics, esters with tertiary amino group (A03AA)

Synthetic anticholinergics, quaternary ammonium compounds (A03AB)
Synthetic antispasmodics, amides with tertiary amines (A03AC)
Synthetic estrogens, plain (G03CB)
Tars (D05AA)
Taxanes (L01CD)
Technetium (99mTc) compounds (V09AA)
Technetium (99mTc) compounds (V09BA)
Technetium (99mTc) compounds (V09CA)
Technetium (99mTc) compounds (V09DA)
Technetium (99mTc) compounds (V09GA)
Technetium (99mTc) compounds (V09HA)
Technetium (99mTc) compounds (V09IA)
Technetium (99mTc), inhalants (V09EA)
Technetium (99mTc), particles and colloids (V09DB)
Technetium (99mTc), particles for injection (V09EB)
Technical disinfectants (V07AV)
Tertiary amines (N04AA)
Testosterone-5-alpha reductase inhibitors (G04CB)
Tests for allergic diseases (V04CL)
Tests for bile duct patency (V04CC)
Tests for diabetes (V04CA)
Tests for fat absorption (V04CB)
Tests for fertility disturbances (V04CM)
Tests for gastric secretion (V04CG)
Tests for liver functional capacity (V04CE)
Tests for pancreatic function (V04CK)
Tests for pituitary function (V04CD)
Tests for renal function and ureteral injuries (V04CH)
Tests for thyreoidea function (V04CJ)
Tetanus vaccines (J07AM)

Tetracycline and derivatives (D06AA)
Tetracyclines (J01AA)
Tetrahydropyrimidine derivatives (P02CC)
Thiazide derivatives (C02DA)
Thiazides and potassium in combination (C03AB)
Thiazides, combinations with other drugs (C03AX)
Thiazides, combinations with psycholeptics and/or analgesics (C03AH)
Thiazides, plain (C03AA)
Thiazolidinediones (A10BG)
Thiocarbamide derivatives (J04AD)
Thiosemicarbazones (J05AA)
Thiouracils (H03BA)
Thioxanthene derivatives (N05AF)
Third-generation cephalosporins (J01DD)
Thyroid hormones (H03AA)
Thyrotropin (H01AB)
Tissue adhesives (V03AK)
Topoisomerase 1 (TOP1) inhibitors (L01CE)
Triazole derivatives (G01AG)
Triazole derivatives (J02AC)
Trimethoprim and derivatives (J01EA)
Tuberculosis diagnostics (V04CF)
Tuberculosis vaccines (J07AN)
Tumor necrosis factor alpha (TNF-a) inhibitors (L04AB)
Typhoid vaccines (J07AP)
Typhus (exanthematicus) vaccines (J07AR)
Tyrosine hydroxylase inhibitors (C02KB)
Ultrasound contrast media (V08DA)
Urinary concrement solvents (G04BC)
Varicella zoster vaccines (J07BK)

Various alimentary tract and metabolism products (A16AX)
Various diagnostic radiopharmaceuticals (V09XX)
Various pain palliation radiopharmaceuticals (V10BX)
Various therapeutic radiopharmaceuticals (V10XX)
Various thyroid diagnostic radiopharmaceuticals (V09FX)
Vascular endothelial growth factor receptor (VEGFR) tyrosine kinase inhibitors (L01EK)
Vasopressin and analogues (H01BA)
Vasopressin antagonists (C03XA)
Vinca alkaloids and analogues (L01CA)
Viscoelastic substances (S01KA)
Vitamin A and D in combination (A11CB)
Vitamin A, plain (A11CA)
Vitamin B1 in combination with vitamin B6 and/or vitamin B12 (A11DB)
Vitamin B1, plain (A11DA)
Vitamin B12 (cyanocobalamin and analogues) (B03BA)
Vitamin B-complex with anabolic steroids (A11ED)
Vitamin B-complex with minerals (A11EC)
Vitamin B-complex with vitamin C (A11EB)
Vitamin B-complex, other combinations (A11EX)
Vitamin B-complex, plain (A11EA)
Vitamin D and analogues (A11CC)
Vitamin K (B02BA)
Vitamin K antagonists (B01AA)
Vitamins (B05XC)
Vitamins with minerals (A11JB)
Vitamins, other combinations (A11JC)
Wart and anti-corn preparations (D11AF)
Washing agents etc. (V07AX)
Watersoluble, hepatotropic X-ray contrast media (V08AC)
Watersoluble, nephrotropic, high osmolar X-ray contrast media (V08AA)

Watersoluble, nephrotropic, low osmolar X-ray contrast media (V08AB)
Xanthine derivatives (C03BD)
Xanthine derivatives (N06BC)
Xanthines (R03DA)
Xanthines and adrenergics (R03DB)
Yellow fever vaccines (J07BL)
Yttrium (90Y) compounds (V10AA)
Zinc (A12CB)
Zinc bandages (D09AB)
Zinc products (D02AB)
