# Supplementary material for: The co-occurrence of multimorbidity and polypharmacy among middle-aged and older adults in Canada: A cross-sectional study using the Canadian Longitudinal Study on Aging (CLSA) and the Canadian Primary Care Sentinel Surveillance Network (CPCSSN)
Source: PLoS One. 2025 Jan 15;20(1):e0312873. doi: 10.1371/journal.pone.0312873 (PMC11734935; doi:10.1371/journal.pone.0312873)
Supplement: S3 Table — (PDF) [file pone.0312873.s003.pdf]

S3 Table. Prevalence of conditions and medications in CLSA and CPCSSN

	CLSA (N = 30097)			CPCSSN (N = 597631)			DIFFERENCE (CLSA-CPCSSN)
**Conditions and Medications**	**n**	**%**	**Order**	**n**	**%**	**Order**	
Hypertension	11096	36.9	1	207649	34.7	3	2.1
Obesity	8933	29.7	2	256399	42.9	1	-13.2
Osteoarthritis or rheumatoid arthritis	8434	28.0	3	82643	13.8	8	14.2
Musculoskeletal problem	8384	27.9	4	231404	38.7	2	-10.9
HMG CoA reductase inhibitors	6320	21.0	5	74593	12.5	10	8.5
Anxiety or depression	6243	20.7	6	189064	31.6	4	-10.9
Diabetes	5307	17.6	7	92954	15.6	6	2.1
Chronic obstructive pulmonary disease or asthma	5093	16.9	8	69575	11.6	11	5.3
Cancer	4636	15.4	9	146710	24.5	5	-9.1
Thyroid problem	4376	14.5	10	58889	9.9	15	4.7
Thyroid hormones	3706	12.3	11	36050	6.0	28	6.3
Proton pump inhibitors	3523	11.7	12	80054	13.4	9	-1.7
Heart failure	3503	11.6	13	10513	1.8	91	9.9
Platelet aggregation inhibitors excl. heparin	3359	11.2	14	33444	5.6	30	5.6
ACE inhibitors, plain	3030	10.1	15	48154	8.1	22	2.0
Cardiovascular disease	2867	9.5	16	58744	9.8	16	-0.3
Osteoporosis	2688	8.9	17	8971	1.5	97	7.4
Urinary problem	2514	8.4	18	63918	10.7	13	-2.3
Stomach problem	2275	7.6	19	31240	5.2	35	2.3
Beta blocking agents, selective	2166	7.2	20	33447	5.6	29	1.6
Angiotensin II receptor blockers (ARBs) and diuretics	2019	6.7	21	388	0.1	324	6.6
Dihydropyridine derivatives	1784	5.9	22	31847	5.3	33	0.6
Other antidepressants	1760	5.8	23	48587	8.1	21	-2.3
Thiazides, plain	1729	5.7	24	27759	4.6	40	1.1
Selective serotonin reuptake inhibitors	1570	5.2	25	55173	9.2	19	-4.0
Stroke or transient ischemic attack	1347	4.5	26	5530	0.9	122	3.6

Natural and semisynthetic estrogens, plain	1148	3.8	27	17514	2.9	56	0.9
Anilides	1117	3.7	28	41022	6.9	23	-3.2
Propionic acid derivatives	1050	3.5	29	56223	9.4	18	-5.9
Angiotensin II receptor blockers (ARBs), other combinations	1047	3.5	30	9439	1.6	96	1.9
Benzodiazepine derivatives	986	3.3	31	59637	10.0	14	-6.7
Adrenergics in combination with corticosteroids or other drugs, excl. anticholinergics	913	3.0	32	31903	5.3	32	-2.3
Salicylic acid and derivatives	907	3.0	33	20569	3.4	45	-0.4
Biguanides	901	3.0	34	31374	5.2	34	-2.3
Selective beta-2-adrenoreceptor agonists	900	3.0	35	64945	10.9	12	-7.9
Kidney disease or failure	866	2.9	36	7262	1.2	103	1.7
Alpha-adrenoreceptor antagonists	823	2.7	37	14418	2.4	65	0.3
Benzodiazepine related drugs	818	2.7	38	29822	5.0	37	-2.3
Other antiepileptics	805	2.7	39	22665	3.8	44	-1.1
Glucocorticoids	759	2.5	40	56990	9.5	17	-7.0
Bisphosphonates	726	2.4	41	17779	3.0	55	-0.6
Corticosteroids	718	2.4	42	87370	14.6	7	-12.2
Sulfonylureas	652	2.2	43	11486	1.9	84	0.2
Coxibs	632	2.1	44	13831	2.3	67	-0.2
Other lipid modifying agents	593	2.0	45	6228	1.0	113	0.9
Colon problem	582	1.9	46	31081	5.2	36	-3.3
ACE inhibitors and diuretics	560	1.9	47	11835	2.0	80	-0.1
Sulfonamides, plain	557	1.9	48	19232	3.2	49	-1.4
Vitamin D and analogues	537	1.8	49	28031	4.7	38	-2.9
Ascorbic acid (vitamin C), plain	523	1.7	50	3072	0.5	157	1.2
Pregnen (4) derivatives	488	1.6	51	6699	1.1	110	0.5
Preparations inhibiting uric acid production	481	1.6	52	6420	1.1	112	0.5
Non-selective monoamine reuptake inhibitors	480	1.6	53	19202	3.2	50	-1.6
Testosterone-5-alpha reductase inhibitors	471	1.6	54	5313	0.9	123	0.7
Drugs for urinary frequency and incontinence	466	1.5	55	7195	1.2	105	0.3

Prostaglandin analogues	463	1.5	56	2704	0.5	171	1.1
Anticholinergics	405	1.3	57	11500	1.9	83	-0.6
Acetic acid derivatives and related substances	397	1.3	58	28006	4.7	39	-3.4
Beta blocking agents	387	1.3	59	2124	0.4	190	0.9
Benzothiazepine derivatives	386	1.3	60	6969	1.2	107	0.1
Insulins and analogues for injection, fast-acting	348	1.2	61	6055	1.0	117	0.1
Insulins and analogues for injection, intermediate- or long-acting combined with fast-acting	322	1.1	62	2017	0.3	195	0.7
Vitamin K antagonists	305	1.0	63	10565	1.8	90	-0.8
Organic nitrates	301	1.0	64	13438	2.2	71	-1.2
Piperazine derivatives	298	1.0	65	11388	1.9	87	-0.9
Natural opium alkaloids	271	0.9	66	32766	5.5	31	-4.6
Dipeptidyl peptidase 4 (DPP-4) inhibitors	265	0.9	67	6114	1.0	115	-0.1
Opioids in combination with non-opioid analgesics	264	0.9	68	16771	2.8	57	-1.9
Corticosteroids, potent (group III)	260	0.9	69	37299	6.2	27	-5.4
Diazepines, oxazepines, thiazepines and oxepines	241	0.8	70	12594	2.1	75	-1.3
Softeners, emollients	239	0.8	71	14735	2.5	63	-1.7
Other antihistamines for systemic use	237	0.8	72	2347	0.4	183	0.4
Low-ceiling diuretics and potassium-sparing agents	233	0.8	73	2612	0.4	176	0.3
Other centrally acting agents	210	0.7	74	19065	3.2	51	-2.5
Beta blocking agents, non-selective	196	0.7	75	4949	0.8	128	-0.2
Direct thrombin inhibitors	194	0.6	76	1175	0.2	238	0.4
Propulsives	188	0.6	77	9798	1.6	95	-1.0
Fibrates	186	0.6	78	2924	0.5	162	0.1
Direct factor Xa inhibitors	183	0.6	79	3094	0.5	154	0.1
Folic acid analogues	183	0.6	80	2507	0.4	178	0.2
Leukotriene receptor antagonists	180	0.6	81	4157	0.7	137	-0.1
Aldosterone antagonists	175	0.6	82	4533	0.8	130	-0.2

Selective serotonin (5HT1) agonists	173	0.6	83	11465	1.9	85	-1.3
3-oxoandrosten (4) derivatives	171	0.6	84	2266	0.4	184	0.2
Drugs used in erectile dysfunction	169	0.6	85	18998	3.2	53	-2.6
Other ophthalmologicals	162	0.5	86	1683	0.3	210	0.3
Folic acid and derivatives	154	0.5	87	5125	0.9	126	-0.3
Aminoquinolines	151	0.5	88	3900	0.7	139	-0.2
Centrally acting sympathomimetics	149	0.5	89	7691	1.3	101	-0.8
Insulins and analogues for injection, intermediate-acting	148	0.5	90	1819	0.3	204	0.2
Contact laxatives	146	0.5	91	13035	2.2	72	-1.7
Nucleosides and nucleotides excl. reverse transcriptase inhibitors	139	0.5	92	20146	3.4	47	-2.9
Aminosalicylic acid and similar agents	138	0.5	93	2667	0.4	173	0.0
Aminoalkyl ethers	129	0.4	94	11404	1.9	86	-1.5
Combinations of oral blood glucose lowering drugs	119	0.4	95	2948	0.5	159	-0.1
Tumor necrosis factor alpha (TNF-a) inhibitors	111	0.4	96	1253	0.2	234	0.2
Dopamine agonists	110	0.4	97	1871	0.3	202	0.1
Alpha and beta blocking agents	105	0.3	98	2395	0.4	181	-0.1
Dopa and dopa derivatives	93	0.3	99	1923	0.3	199	0.0
Other opioids	89	0.3	100	4505	0.8	131	-0.5
Aromatase inhibitors	85	0.3	101	1339	0.2	226	0.1
Progestogens and estrogens, fixed combinations	84	0.3	102	40028	6.7	24	-6.4
Other drugs affecting bone structure and mineralization	81	0.3	103	620	0.1	283	0.2
Glucagon-like peptide-1 (GLP-1) analogues	80	0.3	104	267	0.0	359	0.2
Digitalis glycosides	79	0.3	105	2440	0.4	179	-0.1
Hydantoin derivatives	79	0.3	106	1468	0.2	221	0.0
Tetracyclines	76	0.3	107	14599	2.4	64	-2.2
Bisphosphonates, combinations	76	0.3	108	4999	0.8	127	-0.6
Carbonic anhydrase inhibitors	76	0.3	109	1418	0.2	222	0.0

Corticosteroids, plain	75	0.2	110	740	0.1	269	0.1
Corticosteroids, very potent (group IV)	74	0.2	111	5691	1.0	119	-0.7
Other antipsychotics	73	0.2	112	6125	1.0	114	-0.8
Other antifungals for topical use	71	0.2	113	12051	2.0	78	-1.8
Fatty acid derivatives	71	0.2	114	2781	0.5	167	-0.2
Dementia	68	0.2	115	15823	2.6	60	-2.4
Imidazole and triazole derivatives	67	0.2	116	11692	2.0	82	-1.7
Other chemotherapeutics	67	0.2	117	3985	0.7	138	-0.4
Preparations with no effect on uric acid metabolism	67	0.2	118	3812	0.6	141	-0.4
Carbamic acid esters	67	0.2	119	1404	0.2	223	0.0
Antivertigo preparations	65	0.2	120	6114	1.0	116	-0.8
Selective immunosuppressants	65	0.2	121	1018	0.2	244	0.0
Carboxamide derivatives	64	0.2	122	2023	0.3	194	-0.1
Other antiallergics	63	0.2	123	7945	1.3	100	-1.1
Other antipsoriatics for topical use	63	0.2	124	3681	0.6	144	-0.4
Osmotically acting laxatives	62	0.2	125	12869	2.2	74	-1.9
Lithium	62	0.2	126	1626	0.3	213	-0.1
Vitamin B12 (cyanocobalamin and analogues)	61	0.2	127	5681	1.0	120	-0.7
Anti-estrogens	60	0.2	128	990	0.2	247	0.0
Oxicams	58	0.2	129	11910	2.0	79	-1.8
Anticholinesterases	58	0.2	130	3441	0.6	149	-0.4
H2-receptor antagonists	56	0.2	131	19275	3.2	48	-3.0
Other drugs for functional gastrointestinal disorders	53	0.2	132	1864	0.3	203	-0.1
Other immunosuppressants	51	0.2	133	1245	0.2	235	0.0
Antiarrhythmics, class III	50	0.2	134	1553	0.3	216	-0.1
Angiotensin II receptor blockers (ARBs), plain	49	0.2	135	23764	4.0	43	-3.8
Opium alkaloids and derivatives	49	0.2	136	20392	3.4	46	-3.2
Antiinflammatory preparations, non-steroids for topical use	49	0.2	137	18227	3.0	54	-2.9

HMG CoA reductase inhibitors, other combinations	49	0.2	138	1025	0.2	243	0.0
Insulins and analogues for injection, long-acting	48	0.2	139	7121	1.2	106	-1.0
Bile acid sequestrants	48	0.2	140	1338	0.2	227	-0.1
Antipropulsives	47	0.2	141	3079	0.5	155	-0.4
Methanolquinolines	47	0.2	142	2198	0.4	188	-0.2
Antiarrhythmics, class Ic	47	0.2	143	561	0.1	292	0.1
Calcineurin inhibitors	47	0.2	144	561	0.1	293	0.1
Imidazoline receptor agonists	46	0.2	145	1373	0.2	224	-0.1
Other blood glucose lowering drugs, excl. insulins	46	0.2	146	933	0.2	249	0.0
Selective estrogen receptor modulators	43	0.1	147	416	0.1	316	0.1
Penicillins with extended spectrum	42	0.1	148	38606	6.5	25	-6.3
Diphenylmethane derivatives	39	0.1	149	4456	0.7	133	-0.6
Adrenergic and dopaminergic agents	38	0.1	150	10464	1.8	93	-1.6
Agents for dermatitis, excluding corticosteroids	37	0.1	151	3223	0.5	152	-0.4
Corticosteroids, moderately potent (group II)	37	0.1	152	1651	0.3	212	-0.2
Phenylalkylamine derivatives	36	0.1	153	1081	0.2	241	-0.1
Sympathomimetics in glaucoma therapy	35	0.1	154	668	0.1	277	0.0
Fluoroquinolones	33	0.1	155	37953	6.4	26	-6.2
Corticosteroids and antiinfectives in combination	32	0.1	156	13504	2.3	69	-2.2
Thiazolidinediones	31	0.1	157	1333	0.2	229	-0.1
Barbiturates and derivatives	31	0.1	158	373	0.1	329	0.0
Antivirals for treatment of HIV infections, combinations	30	0.1	159	2015	0.3	196	-0.2
Enzyme preparations	28	0.1	160	748	0.1	265	0.0
Other antibiotics for topical use	27	0.1	161	16063	2.7	59	-2.6
Macrolides	26	0.1	162	53389	8.9	20	-8.8
Hydrazinophthalazine derivatives	25	0.1	163	479	0.1	307	0.0
Antiinflammatory agents, non-steroids	24	0.1	164	2632	0.4	175	-0.4
Other nasal preparations	23	0.1	165	2948	0.5	160	-0.4
Gonadotropin releasing hormone analogues	23	0.1	166	910	0.2	252	-0.1

Bile acids and derivatives	23	0.1	167	323	0.1	344	0.0
Substituted alkylamines	23	0.1	168	287	0.0	351	0.0
Combinations of sulfonamides and trimethoprim, incl. derivatives	22	0.1	169	11723	2.0	81	-1.9
Other dermatologicals	22	0.1	170	3679	0.6	145	-0.5
Monoamine oxidase B inhibitors	22	0.1	171	114	0.0	402	0.1
First-generation cephalosporins	20	0.1	172	27542	4.6	41	-4.5
Antibiotics	20	0.1	173	16260	2.7	58	-2.7
Other antiemetics	20	0.1	174	2005	0.3	197	-0.3
Belladonna alkaloids, semisynthetic, quaternary ammonium compounds	20	0.1	175	1514	0.3	217	-0.2
Sulfur-containing imidazole derivatives	20	0.1	176	661	0.1	278	0.0
Corticosteroids, weak (group I)	19	0.1	177	13926	2.3	66	-2.3
Azaspirodecanedione derivatives	19	0.1	178	508	0.1	301	0.0
Interferons	19	0.1	179	459	0.1	310	0.0
Drugs used in nicotine dependence	18	0.1	180	12929	2.2	73	-2.1
Heparin group	18	0.1	181	1310	0.2	231	-0.2
Beta blocking agents, selective, and other diuretics	18	0.1	182	639	0.1	281	0.0
Antivirals	16	0.1	183	3491	0.6	147	-0.5
Phenothiazines with piperazine structure	16	0.1	184	2818	0.5	166	-0.4
Sympathomimetics	16	0.1	185	741	0.1	268	-0.1
Sodium-glucose co-transporter 2 (SGLT2) inhibitors	16	0.1	186	672	0.1	276	-0.1
Mineralocorticoids	16	0.1	187	240	0.0	365	0.0
Retinoids for topical use in acne	15	0.0	188	4477	0.7	132	-0.7
Heparins or heparinoids for topical use	15	0.0	189	160	0.0	379	0.0
Combinations of penicillins, incl. beta-lactamase inhibitors	14	0.0	190	10386	1.7	94	-1.7
Protease inhibitors	14	0.0	191	793	0.1	261	-0.1
Adamantane derivatives	14	0.0	192	230	0.0	366	0.0
Other immunostimulants	14	0.0	193	130	0.0	394	0.0

Nitrofuran derivatives	13	0.0	194	26449	4.4	42	-4.4
Antifungals for systemic use	13	0.0	195	2058	0.3	193	-0.3
Corticosteroids, moderately potent, combinations with antibiotics	13	0.0	196	1982	0.3	198	-0.3
Corticosteroids, potent, other combinations	13	0.0	197	1004	0.2	246	-0.1
Drugs used in opioid dependence	13	0.0	198	895	0.1	255	-0.1
Vasopressin and analogues	13	0.0	199	406	0.1	318	0.0
Nucleoside and nucleotide reverse transcriptase inhibitors	13	0.0	200	405	0.1	319	0.0
Triazole derivatives	12	0.0	201	13536	2.3	68	-2.2
Phenylpiperidine derivatives	12	0.0	202	2216	0.4	187	-0.3
Pyrimidine analogues	12	0.0	203	748	0.1	266	-0.1
Drugs for treatment of hyperkalemia and hyperphosphatemia	12	0.0	204	449	0.1	312	0.0
Other antineoplastic agents	12	0.0	205	329	0.1	341	0.0
Oripavine derivatives	12	0.0	206	109	0.0	404	0.0
Monoamine oxidase inhibitors, non-selective	12	0.0	207	42	0.0	461	0.0
Vitamins	12	0.0	208	3	0.0	558	0.0
Integrase inhibitors	12	0.0	209	0	0.0		0.0
Intrauterine contraceptives	11	0.0	210	8557	1.4	98	-1.4
Phenothiazines with aliphatic side-chain	11	0.0	211	580	0.1	287	-0.1
Alpha glucosidase inhibitors	11	0.0	212	221	0.0	367	0.0
Other muscle relaxants, peripherally acting agents	10	0.0	213	451	0.1	311	0.0
Other systemic drugs for obstructive airway diseases	10	0.0	214	106	0.0	407	0.0
Detoxifying agents for antineoplastic treatment	10	0.0	215	72	0.0	435	0.0
Imidazole derivatives	9	0.0	216	14817	2.5	62	-2.4
Other drugs for peptic ulcer and gastro-oesophageal reflux disease (GORD)	9	0.0	217	660	0.1	279	-0.1
Non-nucleoside reverse transcriptase inhibitors	9	0.0	218	584	0.1	286	-0.1

Synthetic anticholinergics, esters with tertiary amino group	9	0.0	219	563	0.1	290	-0.1
Purine derivatives	9	0.0	220	393	0.1	323	0.0
Other dopaminergic agents	9	0.0	221	66	0.0	437	0.0
Purine analogues	9	0.0	222	51	0.0	449	0.0
Sympathomimetics used as decongestants	8	0.0	223	3228	0.5	151	-0.5
Serotonin (5HT3) antagonists	8	0.0	224	2184	0.4	189	-0.3
Peripherally acting antiobesity products	8	0.0	225	702	0.1	273	-0.1
Renin-inhibitors	8	0.0	226	470	0.1	308	-0.1
Other drugs for constipation	8	0.0	227	439	0.1	314	0.0
Anti-androgens	8	0.0	228	307	0.1	347	0.0
Parasympathomimetics	8	0.0	229	216	0.0	368	0.0
Other potassium-sparing agents	8	0.0	230	122	0.0	399	0.0
Interleukin inhibitors	8	0.0	231	96	0.0	419	0.0
Bcr-abl tyrosine kinase inhibitors	8	0.0	232	0	0.0		0.0
Progestogens and estrogens, sequential preparations	7	0.0	233	15189	2.5	61	-2.5
Drugs used in alcohol dependence	7	0.0	234	486	0.1	305	-0.1
Indole derivatives	7	0.0	235	282	0.0	353	0.0
Sympathomimetics, plain	7	0.0	236	155	0.0	383	0.0
Antiinfectives for treatment of acne	6	0.0	237	8268	1.4	99	-1.4
Second-generation cephalosporins	6	0.0	238	6825	1.1	109	-1.1
Nitroimidazole derivatives	6	0.0	239	5776	1.0	118	-0.9
Prostaglandins	6	0.0	240	3649	0.6	146	-0.6
Retinoids for treatment of acne	6	0.0	241	1259	0.2	233	-0.2
Antiinfectives and antiseptics for local oral treatment	6	0.0	242	755	0.1	264	-0.1
Other antianemic preparations	6	0.0	243	488	0.1	304	-0.1
Other anti-acne preparations for topical use	6	0.0	244	175	0.0	375	0.0
Monoamine oxidase A inhibitors	6	0.0	245	142	0.0	389	0.0
Parathyroid hormones and analogues	6	0.0	246	64	0.0	438	0.0

Other parasympathomimetics	6	0.0	247	43	0.0	458	0.0
Other agents for local oral treatment	5	0.0	248	7412	1.2	102	-1.2
Corticosteroids, weak, combinations with antibiotics	5	0.0	249	3793	0.6	142	-0.6
Amino acids	5	0.0	250	1881	0.3	201	-0.3
Anesthetics for topical use	5	0.0	251	1514	0.3	218	-0.2
Soft paraffin and fat products	5	0.0	252	1320	0.2	230	-0.2
Iron, parenteral preparations; parenteral	5	0.0	253	332	0.1	339	0.0
Other nervous system drugs	5	0.0	254	104	0.0	411	0.0
Retinoids for treatment of psoriasis	5	0.0	255	102	0.0	413	0.0
Other antiinflammatory and antirheumatic agents, non-steroids	5	0.0	256	95	0.0	421	0.0
Immunoglobulins, normal human	5	0.0	257	94	0.0	422	0.0
Protectives against UV-radiation for systemic use	4	0.0	258	6900	1.2	108	-1.1
Lincosamides	4	0.0	259	5602	0.9	121	-0.9
Antiinfectives	4	0.0	260	1903	0.3	200	-0.3
Corticosteroids acting locally	4	0.0	261	598	0.1	284	-0.1
Other anti-dementia drugs	4	0.0	262	440	0.1	313	-0.1
Ethers, chemically close to antihistamines	4	0.0	263	332	0.1	340	0.0
Antacids with antiflatulents	4	0.0	264	197	0.0	370	0.0
Synthetic anticholinergic agents in combination with analgesics	4	0.0	265	145	0.0	387	0.0
Drugs for treatment of lepra	4	0.0	266	128	0.0	395	0.0
Nitrogen mustard analogues	4	0.0	267	100	0.0	415	0.0
Other analgesics and antipyretics	4	0.0	268	99	0.0	417	0.0
Progestogens	3	0.0	269	6698	1.1	111	-1.1
Trimethoprim and derivatives	3	0.0	270	4546	0.8	129	-0.8
Ergot alkaloids	3	0.0	271	2729	0.5	168	-0.4
Corticosteroids for local oral treatment	3	0.0	272	1184	0.2	237	-0.2
Sulfonamides	3	0.0	273	805	0.1	259	-0.1
Tars	3	0.0	274	550	0.1	296	-0.1

Hemodialytics, concentrates	3	0.0	275	407	0.1	317	-0.1
Prolactine inhibitors	3	0.0	276	267	0.0	360	0.0
Methyldopa	3	0.0	277	180	0.0	373	0.0
Other general anesthetics	3	0.0	278	158	0.0	380	0.0
Somatostatin and analogues	3	0.0	279	89	0.0	425	0.0
Choline esters	3	0.0	280	40	0.0	466	0.0
Phenothiazine derivatives	3	0.0	281	36	0.0	473	0.0
Antigonadotropins and similar agents	3	0.0	282	31	0.0	479	0.0
Other anti-parathyroid agents	3	0.0	283	23	0.0	492	0.0
Beta-lactamase sensitive penicillins	2	0.0	284	11343	1.9	88	-1.9
Hepatitis vaccines	2	0.0	285	11259	1.9	89	-1.9
Third-generation cephalosporins	2	0.0	286	3157	0.5	153	-0.5
Beta-lactamase resistant penicillins	2	0.0	287	2893	0.5	163	-0.5
Antiandrogens and estrogens	2	0.0	288	2888	0.5	164	-0.5
Opium derivatives and expectorants	2	0.0	289	2353	0.4	182	-0.4
Amides	2	0.0	290	1289	0.2	232	-0.2
Butyrophenone derivatives	2	0.0	291	695	0.1	274	-0.1
Antiallergic agents, excl. corticosteroids	2	0.0	292	433	0.1	315	-0.1
Antihistamines for topical use	2	0.0	293	388	0.1	325	-0.1
Electrolyte solutions	2	0.0	294	363	0.1	334	-0.1
Antiarrhythmics, class Ia	2	0.0	295	329	0.1	342	0.0
Antihypertensives for pulmonary arterial hypertension	2	0.0	296	288	0.0	350	0.0
Bismuth preparations	2	0.0	297	244	0.0	363	0.0
Tertiary amines	2	0.0	298	200	0.0	369	0.0
Antineovascularisation agents	2	0.0	299	78	0.0	431	0.0
Other hormone antagonists and related agents	2	0.0	300	51	0.0	450	0.0
Tests for thyreoidea function	2	0.0	301	46	0.0	454	0.0
Pregnadien derivatives	2	0.0	302	40	0.0	467	0.0
Morphinan derivatives	2	0.0	303	21	0.0	496	0.0
Other plain vitamin preparations	1	0.0	304	4363	0.7	134	-0.7

Cholera vaccines	1	0.0	305	4167	0.7	136	-0.7
Silicone products	1	0.0	306	3359	0.6	150	-0.6
Potassium	1	0.0	307	2940	0.5	161	-0.5
Peroxides	1	0.0	308	2236	0.4	185	-0.4
Corticosteroids, weak, other combinations	1	0.0	309	1469	0.2	219	-0.2
Local anesthetics	1	0.0	310	1093	0.2	240	-0.2
Fenamates	1	0.0	311	1006	0.2	245	-0.2
Other urologicals	1	0.0	312	915	0.2	251	-0.1
Biguanides and amidines	1	0.0	313	781	0.1	263	-0.1
Ethers of tropine or tropine derivatives	1	0.0	314	694	0.1	275	-0.1
Allergen extracts	1	0.0	315	400	0.1	321	-0.1
Xanthines	1	0.0	316	373	0.1	330	-0.1
Solutions for parenteral nutrition	1	0.0	317	371	0.1	331	-0.1
Various alimentary tract and metabolism products	1	0.0	318	339	0.1	337	-0.1
Other antivirals	1	0.0	319	337	0.1	338	-0.1
Thioxanthene derivatives	1	0.0	320	283	0.0	352	0.0
Hydrazides	1	0.0	321	280	0.0	355	0.0
Glycogenolytic hormones	1	0.0	322	264	0.0	361	0.0
Vitamin K	1	0.0	323	151	0.0	385	0.0
Enzymes	1	0.0	324	85	0.0	427	0.0
Other drugs for treatment of tuberculosis	1	0.0	325	81	0.0	429	0.0
Dantrolene and derivatives	1	0.0	326	64	0.0	439	0.0
Iron chelating agents	1	0.0	327	45	0.0	455	0.0
Antiarrhythmics, class Ib	1	0.0	328	44	0.0	457	0.0
Colony stimulating factors	1	0.0	329	42	0.0	462	0.0
Other agents against amoebiasis and other protozoal diseases	1	0.0	330	34	0.0	474	0.0
Other surgical aids	1	0.0	331	30	0.0	480	0.0
Somatropin and somatropin agonists	1	0.0	332	27	0.0	486	0.0
Pyrimidine derivatives	1	0.0	333	26	0.0	488	0.0

Gonadotropins	1	0.0	334	22	0.0	494	0.0
Amino acids and derivatives	1	0.0	335	21	0.0	497	0.0
Estren derivatives	1	0.0	336	20	0.0	500	0.0
Aryloxyacetic acid derivatives	1	0.0	337	19	0.0	502	0.0
Other alkylating agents	1	0.0	338	13	0.0	511	0.0
Anthracyclines and related substances	1	0.0	339	12	0.0	514	0.0
Beta blocking agents, non-selective, and other diuretics	1	0.0	340	12	0.0	515	0.0
Local hemostatics	1	0.0	341	10	0.0	518	0.0
Imidazoline derivatives	1	0.0	342	5	0.0	540	0.0
Proteinase inhibitors	1	0.0	343	3	0.0	559	0.0
Anaplastic lymphoma kinase (ALK) inhibitors	1	0.0	344	0	0.0	-	0.0
Drugs used in hereditary angioedema	1	0.0	345	0	0.0	-	0.0
Janus-associated kinase (JAK) inhibitors	1	0.0	346	0	0.0	-	0.0
Other protein kinase inhibitors	1	0.0	347	0	0.0	-	0.0
Retinoids for cancer treatment	1	0.0	348	0	0.0	-	0.0
Iron bivalent, oral preparations	0	0.0	-	19044	3.2	52	-3.2
Varicella zoster vaccines	0	0.0	-	13444	2.2	70	-2.2
Vitamin B-complex, plain	0	0.0	-	12579	2.1	76	-2.1
Calcium	0	0.0	-	12298	2.1	77	-2.1
Multivitamins with minerals	0	0.0	-	10508	1.8	92	-1.8
Calcium, combinations with vitamin D and/or other drugs	0	0.0	-	7249	1.2	104	-1.2
Intravaginal contraceptives	0	0.0	-	5221	0.9	124	-0.9
Vitamins with minerals	0	0.0	-	5145	0.9	125	-0.9
Papillomavirus vaccines	0	0.0	-	4319	0.7	135	-0.7
Bulk-forming laxatives	0	0.0	-	3889	0.7	140	-0.7
Iron in other combinations	0	0.0	-	3737	0.6	143	-0.6
Vitamin B-complex, other combinations	0	0.0	-	3454	0.6	148	-0.6
Magnesium	0	0.0	-	3074	0.5	156	-0.5
Multivitamins, plain	0	0.0	-	3015	0.5	158	-0.5

Phenol and derivatives	0	0.0	-	2841	0.5	165	-0.5
Calcium compounds	0	0.0	-	2713	0.5	169	-0.5
Combinations of vitamins	0	0.0	-	2705	0.5	170	-0.5
Medical gases	0	0.0	-	2686	0.4	172	-0.4
Other topical products for joint and muscular pain	0	0.0	-	2647	0.4	174	-0.4
Combinations for eradication of Helicobacter pylori	0	0.0	-	2604	0.4	177	-0.4
Tuberculosis diagnostics	0	0.0	-	2433	0.4	180	-0.4
Emergency contraceptives	0	0.0	-	2218	0.4	186	-0.4
Vitamins, other combinations	0	0.0	-	2114	0.4	191	-0.4
Corticosteroids for systemic use, combinations	0	0.0	-	2107	0.4	192	-0.4
Other antiinfectives	0	0.0	-	1757	0.3	205	-0.3
Neuraminidase inhibitors	0	0.0	-	1740	0.3	206	-0.3
Preparations containing sulfur	0	0.0	-	1738	0.3	207	-0.3
Tests for diabetes	0	0.0	-	1711	0.3	208	-0.3
Tests for allergic diseases	0	0.0	-	1700	0.3	209	-0.3
Quaternary ammonium compounds	0	0.0	-	1664	0.3	211	-0.3
Antihidrotics	0	0.0	-	1607	0.3	214	-0.3
Acid preparations	0	0.0	-	1572	0.3	215	-0.3
Caries prophylactic agents	0	0.0	-	1469	0.2	220	-0.2
Typhoid vaccines	0	0.0	-	1355	0.2	225	-0.2
Preparations with salicylic acid derivatives	0	0.0	-	1334	0.2	228	-0.2
Pyrethrines, incl. synthetic compounds	0	0.0	-	1225	0.2	236	-0.2
Other diagnostic agents	0	0.0	-	1175	0.2	239	-0.2
Salicylic acid preparations	0	0.0	-	1041	0.2	242	-0.2
Blood transfusion, auxiliary products	0	0.0	-	981	0.2	248	-0.2
Poliomyelitis vaccines	0	0.0	-	931	0.2	250	-0.2
Benzimidazole derivatives	0	0.0	-	905	0.2	253	-0.2
Expectorants	0	0.0	-	900	0.2	254	-0.2
Other antiseptics and disinfectants	0	0.0	-	884	0.1	256	-0.1

Wart and anti-corn preparations	0	0.0	-	875	0.1	257	-0.1
Specific immunoglobulins	0	0.0	-	864	0.1	258	-0.1
Aminosalicylic acid and derivatives	0	0.0	-	805	0.1	260	-0.1
Enemas	0	0.0	-	787	0.1	262	-0.1
Medicated dressings with antiinfectives	0	0.0	-	744	0.1	267	-0.1
Other mineral products	0	0.0	-	740	0.1	270	-0.1
Other antithrombotic agents	0	0.0	-	718	0.1	271	-0.1
Other antimigraine preparations	0	0.0	-	703	0.1	272	-0.1
Vitamin B1, plain	0	0.0	-	656	0.1	280	-0.1
Vitamin A and D in combination	0	0.0	-	628	0.1	282	-0.1
Nicotinic acid and derivatives	0	0.0	-	591	0.1	285	-0.1
Carbohydrates	0	0.0	-	578	0.1	288	-0.1
Influenza vaccines	0	0.0	-	572	0.1	289	-0.1
Other cardiac preparations	0	0.0	-	563	0.1	291	-0.1
Zinc	0	0.0	-	560	0.1	294	-0.1
Pertussis vaccines	0	0.0	-	558	0.1	295	-0.1
Meningococcal vaccines	0	0.0	-	547	0.1	297	-0.1
Vitamin B-complex with vitamin C	0	0.0	-	537	0.1	298	-0.1
Antidiarrheal microorganisms	0	0.0	-	517	0.1	299	-0.1
Estrogens, combinations with other drugs	0	0.0	-	510	0.1	300	-0.1
Carbamide products	0	0.0	-	500	0.1	302	-0.1
Magnesium compounds	0	0.0	-	495	0.1	303	-0.1
Pneumococcal vaccines	0	0.0	-	483	0.1	306	-0.1
Other aminoglycosides	0	0.0	-	467	0.1	309	-0.1
Calcitonin preparations	0	0.0	-	405	0.1	320	-0.1
Other agents for treatment of hemorrhoids and anal fissures for topical use	0	0.0	-	395	0.1	322	-0.1
Bacterial and viral vaccines, combined	0	0.0	-	385	0.1	326	-0.1
Liquid plasters	0	0.0	-	378	0.1	327	-0.1
Other antibacterials	0	0.0	-	374	0.1	328	-0.1
Zinc products	0	0.0	-	367	0.1	332	-0.1

Indifferent preparations	0	0.0	-	366	0.1	333	-0.1
Centrally acting antiobesity products	0	0.0	-	355	0.1	335	-0.1
Capsaicin and similar agents	0	0.0	-	348	0.1	336	-0.1
Corticosteroids, potent, combinations with antibiotics	0	0.0	-	327	0.1	343	-0.1
Colouring agents	0	0.0	-	321	0.1	345	-0.1
Adrenergics in combination with anticholinergics incl. triple combinations with corticosteroids	0	0.0	-	317	0.1	346	-0.1
Antiseptics	0	0.0	-	291	0.0	348	0.0
Intermediate-acting sulfonamides	0	0.0	-	291	0.0	349	0.0
Enzyme and acid preparations, combinations	0	0.0	-	282	0.0	354	0.0
Ascorbic acid (vitamin C), combinations	0	0.0	-	279	0.0	356	0.0
Boric acid products	0	0.0	-	278	0.0	357	0.0
Tetanus vaccines	0	0.0	-	277	0.0	358	0.0
Oral rehydration salt formulations	0	0.0	-	263	0.0	362	0.0
Ovulation stimulants, synthetic	0	0.0	-	242	0.0	364	0.0
Barbiturates, plain	0	0.0	-	197	0.0	371	0.0
Chlorine containing products	0	0.0	-	192	0.0	372	0.0
Blood substitutes and plasma protein fractions	0	0.0	-	180	0.0	374	0.0
Solutions affecting the electrolyte balance	0	0.0	-	165	0.0	376	0.0
Salt solutions	0	0.0	-	162	0.0	377	0.0
Vitamin A, plain	0	0.0	-	161	0.0	378	0.0
Non-selective beta-adrenoreceptor agonists	0	0.0	-	157	0.0	381	0.0
Tuberculosis vaccines	0	0.0	-	157	0.0	382	0.0
Nitrosoureas	0	0.0	-	154	0.0	384	0.0
Mucolytics	0	0.0	-	147	0.0	386	0.0
Xanthine derivatives	0	0.0	-	144	0.0	388	0.0
Other emollients and protectives	0	0.0	-	140	0.0	390	0.0
Yellow fever vaccines	0	0.0	-	140	0.0	391	0.0
Thiouracils	0	0.0	-	136	0.0	392	0.0
Monoclonal antibodies	0	0.0	-	135	0.0	393	0.0

Medicated shampoos	0	0.0	-	126	0.0	396	0.0
Estrogens	0	0.0	-	125	0.0	397	0.0
Synthetic anticholinergics, quaternary ammonium compounds	0	0.0	-	124	0.0	398	0.0
Other hypnotics and sedatives	0	0.0	-	122	0.0	400	0.0
Polymyxins	0	0.0	-	118	0.0	401	0.0
Antivirals for treatment of HCV infections	0	0.0	-	111	0.0	403	0.0
Iodine products	0	0.0	-	109	0.0	405	0.0
ACE inhibitors and calcium channel blockers	0	0.0	-	108	0.0	406	0.0
Selenium	0	0.0	-	106	0.0	408	0.0
Combinations and complexes of aluminium, calcium and magnesium compounds	0	0.0	-	105	0.0	409	0.0
Vitamin B1 in combination with vitamin B6 and/or vitamin B12	0	0.0	-	105	0.0	410	0.0
Guanidine derivatives	0	0.0	-	104	0.0	412	0.0
Aldehydes and derivatives	0	0.0	-	101	0.0	414	0.0
Antiandrogens, plain	0	0.0	-	100	0.0	416	0.0
Tetrahydropyrimidine derivatives	0	0.0	-	99	0.0	418	0.0
Sympathomimetics, combinations excl. corticosteroids	0	0.0	-	96	0.0	420	0.0
Rabies vaccines	0	0.0	-	94	0.0	423	0.0
Solvents and diluting agents, incl. irrigating solutions	0	0.0	-	92	0.0	424	0.0
Vitamin B-complex with minerals	0	0.0	-	87	0.0	426	0.0
Other viral vaccines	0	0.0	-	83	0.0	428	0.0
Other antipruritics	0	0.0	-	80	0.0	430	0.0
Analgesics and anesthetics	0	0.0	-	75	0.0	432	0.0
Other ectoparasiticides, incl. scabicides	0	0.0	-	75	0.0	433	0.0
Quinoline derivatives and related substances	0	0.0	-	75	0.0	434	0.0
Belladonna alkaloids, tertiary amines	0	0.0	-	70	0.0	436	0.0
Sodium	0	0.0	-	64	0.0	440	0.0

Fat/carbohydrates/proteins/minerals/vitamins, combinations	0	0.0	-	62	0.0	441	0.0
Blood coagulation factors	0	0.0	-	59	0.0	442	0.0
Progesterone receptor modulators	0	0.0	-	59	0.0	443	0.0
Opioid anesthetics	0	0.0	-	58	0.0	444	0.0
Other intestinal adsorbents	0	0.0	-	58	0.0	445	0.0
Antacids with sodium bicarbonate	0	0.0	-	54	0.0	446	0.0
Esters of aminobenzoic acid	0	0.0	-	54	0.0	447	0.0
Iron in combination with folic acid	0	0.0	-	52	0.0	448	0.0
Carbapenems	0	0.0	-	51	0.0	451	0.0
Glycopeptide antibacterials	0	0.0	-	51	0.0	452	0.0
Quinoline derivatives	0	0.0	-	47	0.0	453	0.0
Benzomorphan derivatives	0	0.0	-	45	0.0	456	0.0
Antidotes	0	0.0	-	43	0.0	459	0.0
Diphenylbutylpiperidine derivatives	0	0.0	-	43	0.0	460	0.0
Charcoal preparations	0	0.0	-	42	0.0	463	0.0
Preparations increasing uric acid excretion	0	0.0	-	42	0.0	464	0.0
Barium sulfate containing X-ray contrast media	0	0.0	-	41	0.0	465	0.0
Watersoluble, nephrotropic, high osmolar X-ray contrast media	0	0.0	-	40	0.0	468	0.0
Soft paraffin dressings	0	0.0	-	39	0.0	469	0.0
Other antiinfectives and antiseptics	0	0.0	-	38	0.0	470	0.0
Other local anesthetics	0	0.0	-	38	0.0	471	0.0
Rauwolfia alkaloids	0	0.0	-	38	0.0	472	0.0
Measles vaccines	0	0.0	-	34	0.0	475	0.0
Diphenylpropylamine derivatives	0	0.0	-	33	0.0	476	0.0
Other combinations of nutrients	0	0.0	-	33	0.0	477	0.0
Taxanes	0	0.0	-	33	0.0	478	0.0
Organic acids	0	0.0	-	30	0.0	481	0.0
Platinum compounds	0	0.0	-	30	0.0	482	0.0
Serotonin receptor antagonists	0	0.0	-	30	0.0	483	0.0

Encephalitis vaccines	0	0.0	-	29	0.0	484	0.0
Halogenated hydrocarbons	0	0.0	-	28	0.0	485	0.0
Hydroxyquinoline derivatives	0	0.0	-	27	0.0	487	0.0
Other bacterial vaccines	0	0.0	-	25	0.0	489	0.0
Oxazol, thiazine, and triazine derivatives	0	0.0	-	25	0.0	490	0.0
Corticosteroids, combinations for treatment of acne	0	0.0	-	24	0.0	491	0.0
Iron trivalent, oral preparations	0	0.0	-	23	0.0	493	0.0
Antispasmodics in combination with other drugs	0	0.0	-	22	0.0	495	0.0
Combinations of drugs for treatment of tuberculosis	0	0.0	-	21	0.0	498	0.0
Lung surfactants	0	0.0	-	21	0.0	499	0.0
Other agents against leishmaniasis and trypanosomiasis	0	0.0	-	20	0.0	501	0.0
Succinimide derivatives	0	0.0	-	19	0.0	503	0.0
Gold preparations	0	0.0	-	18	0.0	504	0.0
Aluminium agents	0	0.0	-	17	0.0	505	0.0
Other cough suppressants	0	0.0	-	17	0.0	506	0.0
Other therapeutic products	0	0.0	-	17	0.0	507	0.0
Vinca alkaloids and analogues	0	0.0	-	15	0.0	508	0.0
Penicillamine and similar agents	0	0.0	-	14	0.0	509	0.0
Phenothiazines with piperidine structure	0	0.0	-	14	0.0	510	0.0
Antacids, other combinations	0	0.0	-	13	0.0	512	0.0
Papaverine and derivatives	0	0.0	-	13	0.0	513	0.0
Peripheral opioid receptor antagonists	0	0.0	-	12	0.0	516	0.0
Aluminium compounds	0	0.0	-	11	0.0	517	0.0
Other respiratory system products	0	0.0	-	10	0.0	519	0.0
Piperazine and derivatives	0	0.0	-	10	0.0	520	0.0
Rubella vaccines	0	0.0	-	10	0.0	521	0.0
Respiratory stimulants	0	0.0	-	9	0.0	522	0.0

Sensitizers used in photodynamic/radiation therapy	0	0.0	-	9	0.0	523	0.0
Tests for bile duct patency	0	0.0	-	9	0.0	524	0.0
Drugs for treatment of hypercalcemia	0	0.0	-	8	0.0	525	0.0
Sclerosing agents for local injection	0	0.0	-	8	0.0	526	0.0
Sympathomimetics excl. antiglaucoma preparations	0	0.0	-	8	0.0	527	0.0
Alpha- and beta-adrenoreceptor agonists	0	0.0	-	7	0.0	528	0.0
Corticosteroids, moderately potent, other combinations	0	0.0	-	7	0.0	529	0.0
Ethers chemically close to antihistamines	0	0.0	-	7	0.0	530	0.0
Podophyllotoxin derivatives	0	0.0	-	7	0.0	531	0.0
Tests for gastric secretion	0	0.0	-	7	0.0	532	0.0
Watersoluble, nephrotropic, low osmolar X-ray contrast media	0	0.0	-	7	0.0	533	0.0
Iodine therapy; systemic	0	0.0	-	6	0.0	534	0.0
Other antimycotics for systemic use	0	0.0	-	6	0.0	535	0.0
Other cytotoxic antibiotics	0	0.0	-	6	0.0	536	0.0
Phosphodiesterase inhibitors	0	0.0	-	6	0.0	537	0.0
Ultrasound contrast media	0	0.0	-	6	0.0	538	0.0
Urinary concrement solvents	0	0.0	-	6	0.0	539	0.0
Amino acids, incl. combinations with polypeptides	0	0.0	-	5	0.0	541	0.0
Anti-gonadotropin-releasing hormones	0	0.0	-	5	0.0	542	0.0
Antidepressants in combination with psycholeptics	0	0.0	-	5	0.0	543	0.0
Corticosteroids, moderately potent, combinations with antiseptics	0	0.0	-	5	0.0	544	0.0
Diaminopyrimidines	0	0.0	-	5	0.0	545	0.0
Other antinematodals	0	0.0	-	5	0.0	546	0.0
Other cough suppressants and expectorants	0	0.0	-	5	0.0	547	0.0
Other non-therapeutic auxiliary products	0	0.0	-	5	0.0	548	0.0

Rota virus diarrhea vaccines	0	0.0	-	5	0.0	549	0.0
Sympathomimetics, labour repressants	0	0.0	-	5	0.0	550	0.0
ACTH	0	0.0	-	4	0.0	551	0.0
Androgens and estrogens	0	0.0	-	4	0.0	552	0.0
Gonadotropin-releasing hormones	0	0.0	-	4	0.0	553	0.0
Milk substitutes	0	0.0	-	4	0.0	554	0.0
Other cicatrizants	0	0.0	-	4	0.0	555	0.0
Rauwolfia alkaloids and diuretics in combination	0	0.0	-	4	0.0	556	0.0
Tests for pituitary function	0	0.0	-	4	0.0	557	0.0
Anesthetics, local	0	0.0	-	3	0.0	560	0.0
Antracen derivatives	0	0.0	-	3	0.0	561	0.0
Barbiturates, combinations	0	0.0	-	3	0.0	562	0.0
Butylpyrazolidines	0	0.0	-	3	0.0	563	0.0
Diphtheria vaccines	0	0.0	-	3	0.0	564	0.0
Immune sera	0	0.0	-	3	0.0	565	0.0
Other quaternary ammonium compounds	0	0.0	-	3	0.0	566	0.0
Other systemic hemostatics	0	0.0	-	3	0.0	567	0.0
Paramagnetic contrast media	0	0.0	-	3	0.0	568	0.0
Proteolytic enzymes	0	0.0	-	3	0.0	569	0.0
Watersoluble, hepatotropic X-ray contrast media	0	0.0	-	3	0.0	570	0.0
Amino acids/carbohydrates/minerals/vitamins, combinations	0	0.0	-	2	0.0	571	0.0
Carbamates	0	0.0	-	2	0.0	572	0.0
Combinations of antibacterials	0	0.0	-	2	0.0	573	0.0
Haemophilus influenzae B vaccines	0	0.0	-	2	0.0	574	0.0
Non-watersoluble X-ray contrast media	0	0.0	-	2	0.0	575	0.0
Other anterior pituitary lobe hormones and analogues	0	0.0	-	2	0.0	576	0.0
Other antimalarials	0	0.0	-	2	0.0	577	0.0
Other psychostimulants and nootropics	0	0.0	-	2	0.0	578	0.0
Oxytocin and analogues	0	0.0	-	2	0.0	579	0.0

Psoralens for topical use	0	0.0	-	2	0.0	580	0.0
Thiazide derivatives	0	0.0	-	2	0.0	581	0.0
Washing agents etc.	0	0.0	-	2	0.0	582	0.0
2-amino-1-phenylethanol derivatives	0	0.0	-	1	0.0	583	0.0
Drugs for treatment of hypoglycemia	0	0.0	-	1	0.0	584	0.0
Fluoride	0	0.0	-	1	0.0	585	0.0
HMG CoA reductase inhibitors in combination with other lipid modifying agents	0	0.0	-	1	0.0	586	0.0
Hypertonic solutions	0	0.0	-	1	0.0	587	0.0
Imidazothiazole derivatives	0	0.0	-	1	0.0	588	0.0
Interleukins	0	0.0	-	1	0.0	589	0.0
Iodine (131I) compounds	0	0.0	-	1	0.0	590	0.0
Nitroferricyanide derivatives	0	0.0	-	1	0.0	591	0.0
Other i.v. solution additives	0	0.0	-	1	0.0	592	0.0
Other irrigating solutions	0	0.0	-	1	0.0	593	0.0
Preparations for biliary tract therapy	0	0.0	-	1	0.0	594	0.0
Steroid antibacterials	0	0.0	-	1	0.0	595	0.0
Synthetic estrogens, plain	0	0.0	-	1	0.0	596	0.0
Tests for pancreatic function	0	0.0	-	1	0.0	597	0.0
Vasopressin antagonists	0	0.0	-	1	0.0	598	0.0
Zinc bandages	0	0.0	-	1	0.0	599	0.0
