# Supplementary material for: The co-occurrence of multimorbidity and polypharmacy among middle-aged and older adults in Canada: A cross-sectional study using the Canadian Longitudinal Study on Aging (CLSA) and the Canadian Primary Care Sentinel Surveillance Network (CPCSSN)
Source: PLoS One. 2025 Jan 15;20(1):e0312873. doi: 10.1371/journal.pone.0312873 (PMC11734935; doi:10.1371/journal.pone.0312873)
Supplement: Attachment — Submitted filename: MM and PP Manuscript_Response To Reviewers.docx [file pone.0312873.s004.docx]

| **Journal Requirements:** | **Responses:** |
| --- | --- |
| 1. When submitting your revision, we need you to address these additional requirements.  Please ensure that your manuscript meets PLOS ONE's style requirements, including those for file naming. The PLOS ONE style templates can be found at  <https://journals.plos.org/plosone/s/file?id=wjVg/PLOSOne_formatting_sample_main_body.pdf> and <https://journals.plos.org/plosone/s/file?id=ba62/PLOSOne_formatting_sample_title_authors_affiliations.pdf> | The style requirements were reviewed and updated as requested. |
| 2. Thank you for stating in your Funding Statement:  This study was supported by the Canadian Institutes of Health Research Priority Announcement in Aging (175365).  This funding source had no role in the conduct of this study, the writing of the manuscript or the decision to submit for publication.  Please provide an amended statement that declares all the funding or sources of support (whether external or internal to your organization) received during this study, as detailed online in our guide for authors at <http://journals.plos.org/plosone/s/submit-now>.  Please also include the statement “There was no additional external funding received for this study.” in your updated Funding Statement.  Please include your amended Funding Statement within your cover letter. We will change the online submission form on your behalf. | The funding statement was updated as requested. |
| 3. Thank you for stating the following in the Acknowledgments Section of your manuscript:  This study was made possible using the data/biospecimens collected by the Canadian Longitudinal Study on Aging (CLSA) and the Canadian Primary Care Sentinel Surveillance Network (CPCSSN).  Funding for the CLSA is provided by the Government of Canada through the Canadian Institutes of Health Research (CIHR) under grant reference: LSA 94473 and the Canada Foundation for Innovation, as well as the following provinces, Newfoundland, Nova Scotia, Quebec, Ontario, Manitoba, Alberta, and British Columbia.  This study was conducted using the CLSA Baseline Comprehensive Dataset Version 6.0 under Application Number 2104064 and the CPCSSN Dataset under Application Number 2017SRSC99.  The CLSA is led by Drs. Parminder Raina, Christina Wolfson and Susan Kirkland.  The research ethics approval was obtained from the Hamilton Integrated Research Ethics Board (HiREB) Project Number 3586.  This study was supported by the Canadian Institutes of Health Research Priority Announcement in Aging (175365).  This funding source had no role in the conduct of this study, the writing of the manuscript or the decision to submit for publication.  There were no other funding sources for this research.  We note that you have provided funding information that is not currently declared in your Funding Statement. However, funding information should not appear in the Acknowledgments section or other areas of your manuscript. We will only publish funding information present in the Funding Statement section of the online submission form.  Please remove any funding-related text from the manuscript and let us know how you would like to update your Funding Statement. Currently, your Funding Statement reads as follows:  This study was supported by the Canadian Institutes of Health Research Priority Announcement in Aging (175365).  This funding source had no role in the conduct of this study, the writing of the manuscript or the decision to submit for publication.  Please include your amended statements within your cover letter; we will change the online submission form on your behalf. | The acknowledgements section was updated as requested. |
| 4. Thank you for uploading your study's underlying data set. Unfortunately, the repository you have noted in your Data Availability statement does not qualify as an acceptable data repository according to PLOS's standards.  At this time, please upload the minimal data set necessary to replicate your study's findings to a stable, public repository (such as figshare or Dryad) and provide us with the relevant URLs, DOIs, or accession numbers that may be used to access these data. For a list of recommended repositories and additional information on PLOS standards for data deposition, please see <https://journals.plos.org/plosone/s/recommended-repositories>. | Unfortunately, the data cannot be reposited as requested based on the guidelines of the CLSA and CPCSSN datasets, but the Data Availability Statement outlines the process for obtaining access to the respective datasets. |
| 5. PLOS requires an ORCID iD for the corresponding author in Editorial Manager on papers submitted after December 6th, 2016. Please ensure that you have an ORCID iD and that it is validated in Editorial Manager. To do this, go to ‘Update my Information’ (in the upper left-hand corner of the main menu), and click on the Fetch/Validate link next to the ORCID field. This will take you to the ORCID site and allow you to create a new iD or authenticate a pre-existing iD in Editorial Manager. Please see the following video for instructions on linking an ORCID iD to your Editorial Manager account: <https://www.youtube.com/watch?v=_xcclfuvtxQ> | The ORCID iD for the corresponding authors was added as requested. |
| 6. Your ethics statement should only appear in the Methods section of your manuscript. If your ethics statement is written in any section besides the Methods, please delete it from any other section. | The ethics statement was included in the Methods section as requested. |
| 7. Please remove your figures from within your manuscript file, leaving only the individual TIFF/EPS image files, uploaded separately. These will be automatically included in the reviewers’ PDF. | The figures were uploaded separately as requested. |
| 8. Please review your reference list to ensure that it is complete and correct. If you have cited papers that have been retracted, please include the rationale for doing so in the manuscript text, or remove these references and replace them with relevant current references. Any changes to the reference list should be mentioned in the rebuttal letter that accompanies your revised manuscript. If you need to cite a retracted article, indicate the article’s retracted status in the References list and also include a citation and full reference for the retraction notice. | The reference list was reviewed as requested. |
| **Reviewer #1:** |  |
| First, concerning the abstract, the maximum permitted length is 300 words (provided with ~ 380). | The abstract has been updated as requested. |
| Second, as stated in the data resource profile of the CPCSSN (doi: 10.1093/ije/dyw248) “… older adults and females are over-represented in the CPCSSN database as compared with the national population. As such it is important to consider age and sex standardization and/or adjustment for all surveillance and research studies employing these data”, it is advisable also to report the age- and sex-standardized values of the overall prevalence of the co-occurrence of MM and PP (and each in isolation) for this dataset, or to state the reason(s) for the exclusion of this step. | The prevalence of the co-occurrence of multimorbidity and polypharmacy has been presented stratified between age groups (middle aged and older adults) and between females and males. |
| **Reviewer #2:** |  |
| 1- The abstract is relatively long and could be more concise, particularly in the results section, to enhance readability and clarity. | The abstract has been updated as requested. |
| 2- Although the authors stated that they conducted a descriptive study, a statistical comparison (e.g., Chi-squared test in Table 1) could be useful for better understanding the differences between the two cohorts. | The statistical comparison in Table 1 was provided as requested. |
| 3- The discussion section could be strengthened by addressing the following points:  A. The reasons for the differences in the prevalence of poly-pharmacy and multi-morbidity between studies.  B. The clinical consequences and health impacts of poly-pharmacy and multi-morbidity should be addressed.  C. Discussing the influence of factors such as socioeconomic status and lifestyle on poly-pharmacy and multi-morbidity can enhance understanding of the topic and the significance of the study.  D. The discussion should align with the results and emphasize the significance and effectiveness of such studies. | The discussion section was updated with these details as requested. |
